# Microbial community coalescence restructures co-occurrence architecture and generates regime-specific patterns of network complexity and stability

**DOI:** 10.1093/ismeco/ycag260

**Published:** 2026-09-12

**Authors:** Luana Bresciani, Gordon F Custer, Francisco Dini-Andreote

**Affiliations:** Department of Plant Science and Huck Institutes of the Life Sciences, The Pennsylvania State University, University Park, PA, 16802, United States; The One Health Microbiome Center, Huck Institutes of the Life Sciences, The Pennsylvania State University, University Park, PA, 16802, United States; Department of Plant Science and Huck Institutes of the Life Sciences, The Pennsylvania State University, University Park, PA, 16802, United States; The One Health Microbiome Center, Huck Institutes of the Life Sciences, The Pennsylvania State University, University Park, PA, 16802, United States; Department of Natural Sciences, School of Agriculture and Natural Resources, The University of Maryland Eastern Shore, Princess Anne, MD, 21853, United States; Department of Plant Science and Huck Institutes of the Life Sciences, The Pennsylvania State University, University Park, PA, 16802, United States; The One Health Microbiome Center, Huck Institutes of the Life Sciences, The Pennsylvania State University, University Park, PA, 16802, United States

**Keywords:** microbial community coalescence, co-occurrence networks, community assembly, network stability, microbiome architecture, microbial diversity

## Abstract

Microbial community coalescence (MCC), the mixing of distinct microbiomes, is increasingly recognized as an important process in natural and managed microbial systems. However, how MCC reorganizes species association architecture and whether this restructuring alters community stability remain poorly understood. Here, we investigated how MCC modifies microbial co-occurrence networks and whether resulting architectures deviate from expectations based on stochastic reshuffling or direct compositional mixing of donor communities. Using controlled soil microcosms, we manipulated pairwise coalescence scenarios across a gradient of biotic dilution and tracked community reassembly over 30 days. Empirical association networks were then compared to two reference frameworks: a stochastic balanced-averaging null model and a donor-preserving compositional mixing model. Across treatments, MCC produced extensive restructuring of species association architecture, with empirical networks exhibiting substantially higher node turnover and a predominance of novel correlations relative to both reference expectations. Higher-order network properties also frequently diverged from expected simulated outcomes, indicating that coalescence reshapes correlation architecture beyond stochastic or additive donor mixing. The consequences of this restructuring for network stability were strongly context-dependent. In one coalescence regime, reductions in biotic complexity led to coordinated declines in network complexity and robustness, accompanied by increased fragmentation and vulnerability, whereas other regimes remained insensitive to the biotic dilution gradient. Moreover, community diversity predicted network architecture only under specific coalescence contexts, revealing regime-specific relationships between community diversity, network complexity, and network stability. Together, these results demonstrate that MCC reorganizes microbial association networks in patterns consistent with context-dependent ecological processes that generate distinct complexity and stability patterns.

## Introduction

Microbial community coalescence (MCC), defined as the mixture of entire microbial communities and their abiotic environments, is a pervasive process in natural and managed ecosystems [1]. MCC occurs across diverse contexts, including river confluences or flooding events in aquatic systems, the incorporation of organic amendments in agricultural soils, and fecal microbiota transplantation in clinical settings [2–4]. By simultaneously merging biotic assemblages and environmental matrices, MCC results in a dynamic rearrangement of biotic and abiotic filters that are thought to jointly modulate the structure and function of the resulting community [5, 6]. Importantly, community reassembly following coalescence is not governed exclusively by environmental filtering or biotic interactions, but rather by their continuous interplay, with the relative importance of each process varying across ecological contexts. For example, stringent abiotic conditions can promote persistence of pre-adapted microorganisms through “home-field advantage” mechanisms [7], whereas in environments with less stringent abiotic filters, biotic interactions may exert a stronger influence on community reorganization [8].

Given the inherent difficulty of directly quantifying species interactions in complex microbial communities, correlation-based co-occurrence networks have been widely used to characterize patterns of taxon co-variation [9–11]. In these networks, nodes represent taxa and edges represent statistically significant positive or negative correlations in their relative abundances across samples. Because such correlations may arise from shared environmental responses, compositional constraints, indirect associations, or true biological interactions, they do not provide direct evidence of ecological interactions and must therefore be interpreted cautiously. Nevertheless, as descriptive representations of community organization, co-occurrence networks enable the quantification of multiple complementary topological parameters (e.g. network size, connectivity, clustering coefficient, modularity, nestedness, and cohesion) that collectively characterize patterns of species associations. These metrics do not identify specific mechanisms but allow for systematic comparison of community organization and may reflect the combined influence of biotic interactions and environmental filtering modulating assemblage structure [11–14]. For example, a recent study on MCC showed that mixing distinct source communities can alter network structural properties, potentially increasing co-occurrence network complexity through the formation of novel associations among previously non-correlated taxa [15].

Changes in species association architecture, i.e. the patterns of species co-occurrence, following MCC may have important consequences for community-level stability. Network analyses indicate that patterns of connectivity, modularity, and the distribution of links among taxa can influence how systems respond to perturbation [16]. For instance, competitive interactions and resource differentiation have been shown to promote coexistence under certain architectural configurations, thereby contributing to structural stability [16, 17]. Within co-occurrence frameworks, stability is commonly assessed through node removal simulations that evaluate network robustness (persistence of nodes following random or targeted removal), fragmentation (degree of network disconnection), and vulnerability (change in global network efficiency after removal of highly connected nodes) [14, 18, 19]. These simulations do not measure ecological resilience directly but rather quantify the structural sensitivity of networks to perturbation. For example, it has been shown that the removal of highly connected “hub” nodes or inter-module connectors can disproportionately reduce connectivity and promote rapid compositional shifts and network collapse [14, 20]. Moreover, other empirical studies have reported positive associations between certain metrics of network complexity, including increased size, link density, and compartmentalization, and enhanced structural robustness [21], while greater community diversity has also been linked to more stable network configurations through increased species-complementary interactions rather than direct competition (e.g. niche differentiation, resource partitioning) [22].

In a previous study, we demonstrated that microbial community reassembly following MCC is modulated by a dynamic context-dependent interplay between environmental filtering and biotic interactions [7]. Here, we build upon the same experimental system and dataset to extend this framework to investigate how MCC reorganizes species co-occurrence networks and how such reorganization influences emergent network architecture, complexity, and structural stability. Using a controlled microcosm experiment with three soils originating from distinct systems, we manipulated initial community composition through a dilution-to-extinction approach to generate contrasting levels of biotic complexity. Pairwise MCC events were conducted by mixing soils at matched dilution levels, and community reassembly dynamics were monitored over 30 days following MCC (Days 1, 5, 15, and 30). To determine whether observed network properties deviate from expectations derived from compositional processes alone, we compared empirical post-coalescence networks against two complementary reference frameworks: (i) a stochastic balanced-averaging null model (BANM), representing randomized reshuffling of donor community composition prior to averaging, and (ii) a donor-preserving balanced donor-sum (BDS) model, representing direct taxonomic blending. Specifically, we sought to answer the following questions: (i) how does MCC restructure species association patterns and correlation architecture relative to donor communities and reference expectations? (ii) Does MCC modify network complexity and structural stability beyond stochastic reshuffling and additive compositional blending? And (iii) to what extent do the donor biotic complexity and variation in the outcome community diversity explain changes in emergent network topological properties across coalescence regimes?

## Materials and methods

### Soil collection and community manipulation

Soil samples were collected, and community structure was manipulated as previously described in Bresciani *et al.* [7]. Briefly, soils were sampled from three distinct fields (hereafter referred to as soils A, B, and C), selected based on their relatively low clay content and anticipated differences in chemical and biological properties. A subset of each soil sample was sterilized via gamma irradiation (>35 kGy) at the Penn State University Gamma Irradiation Facility (Cobalt-60 Pool Irradiator), whereas the remaining non-sterilized soils were stored at 4°C and used as inoculum sources. Sterilized soils A, B, and C were subsequently reinoculated with their respective microbial communities subjected to serial dilution. Specifically, 10 g of each natural soil were serially diluted in sterile deionized (DI) water (1:10 dilution series), and three biotic dilution levels (10^−1^, 10^−3^, and 10^−5^) were used for independent inoculations. Inoculations were performed in three replicate bags per treatment to capture recolonization variability, with 10 ml of diluted inoculum added to 100 g of sterile soil. Soil moisture was adjusted to 65% water holding capacity (WHC) using sterile DI water, and reinoculated soils were incubated at 25°C for 60 days. Following incubation, replicate bags within each soil type and dilution level were homogenized and re-incubated for an additional 7 days to allow further community stabilization [7]. This step was performed to reduce stochastic variation among replicate inoculation bags within each soil × dilution treatment, particularly at higher dilution levels where stochastic drift is expected to be stronger. The additional incubation allowed the homogenized communities to stabilize before initiating the coalescence experiment, thereby providing more consistent donor inocula for subsequent manipulation.

### Experimental design and sample collection

All possible pairwise combinations of MCC were performed by mixing distinct soils at matched dilution levels, as described previously [7]. For each coalescence event, 12.5 g of each donor soil were combined using a sterile spatula and vortexed at maximum speed for 30 s to ensure thorough mixing. Controls consisted of 25 g of each individual donor soil at each corresponding dilution level. Microcosms were incubated at 25°C in the dark for 30 days and covered with sterile cotton to allow gas exchange while minimizing contamination. Soil moisture was maintained at 65% WHC and checked every 5 days, with adjustments made as necessary. In total, the experiment comprised 432 independent microcosms, calculated as follows: [3 soil pairs × 3 biotic dilution levels × 4 sampling time points × 6 replicates] + [3 soil controls × 3 biotic dilution levels × 4 sampling time points × 6 replicates]. To assess temporal community dynamics, microcosms were destructively sampled on Days 1, 5, 15, and 30 following MCC. Throughout the manuscript, treatment sets are denoted using soil identifiers (A, B, and C), MCC combinations (AxB, AxC, and BxC), and biotic dilution levels (10^−1^, 10^−3^, and 10^−5^).

### Soil DNA extraction and sequencing

Soil DNA was extracted using the DNeasy PowerSoil Pro Kit (QIAGEN, USA) according to the manufacturer’s instructions. DNA concentration and purity were assessed using a NanoDrop One Microvolume UV–Vis spectrophotometer (Thermo Fisher Scientific, USA) by measuring absorbance at A260/A280. Extracted DNA was stored at −80°C until further processing. V4 region of the 16S rRNA gene amplicons was amplified using the primers 515F (5′-GTG YCA GCM GCC GCG GTA A-3′) [23] and 806R (5′-GGA CTA CNV GGG TWT CTA AT-3′) [24]. PCR amplification reactions (25 μl total volume) consisted of 0.25 μl Phusion High-Fidelity DNA Polymerase, 5 μl 5× Phusion Green HF buffer, 0.5 μl deoxynucleoside triphosphates (dNTPs; 10 mM), 17.75 μl diethyl pyrocarbonate-treated water, 0.25 μl of each forward and reverse primer (10 μM), and 1 μl of template DNA (approximately 10 ng). Thermocycling conditions were as follows: initial denaturation at 94°C for 3 min; 30 cycles of 94°C for 45 s, 50°C for 60 s, and 72°C for 90 s; followed by a final extension at 72°C for 10 min. PCR products were verified by electrophoresis on 1.5% agarose gels stained with SYBR Safe DNA Gel Stain (Invitrogen, USA). Amplicons were sequenced on an Illumina MiSeq platform using the standard V2 reagent kit (2 × 250 bp paired-end sequencing) at the Penn State University Genomics Core Facility.

### Amplicon sequencing analysis

Raw sequences were demultiplexed, and reads were quality filtered and trimmed using the DADA2 package (v1.20) [25] in R (v4.4.1) [26]. Forward and reverse reads were truncated at 240 and 220 base pairs, respectively, using the filterAndTrim function with the following parameters: truncLen = c(240, 220), maxEE = c(2, 2), truncQ = 2, maxN = 0, and rm.phix = TRUE. Filtered paired-end reads were subsequently merged, and downstream processing was conducted in QIIME 2 (v 2023.7) [27]. Sequences were dereplicated, clustered into operational taxonomic units (OTUs) at 97% sequence identity using the vsearch plugin within QIIME 2, and subsequently screened for chimeras [28]. Taxonomic classification was assigned using a naïve Bayes classifier trained on the SILVA v138 reference database [29] via the QIIME 2 feature-classifier plugin [30]. OTUs not assigned to Bacteria, as well as those classified as Mitochondria or Chloroplast, were removed prior to downstream analyses. OTUs with fewer than four total reads across the dataset and < 0.01% relative abundance within each treatment were excluded from further analysis. The resulting OTU table was used for subsequent ecological analyses, and donor datasets were used to construct null expectations and perform additive taxa mixing simulations, as described below.

### Balanced averaging null model (BANM) simulation

To evaluate whether empirical MCC outcomes deviated from null expectations, we implemented a permutation-based balanced averaging null model (BANM). This framework randomizes donor pairings while enforcing equal contributions from each donor, thereby generating stochastic mixture expectations in the absence of deterministic donor-driven reassembly. Using the donor OTU abundance matrices, pairwise donor mixtures (Donor X and Donor Y) were simulated in two complementary directions: (i) each replicate of Donor X was combined with randomly selected replicates from Donor Y, and (ii) each replicate of Donor Y was combined with randomly selected replicates from Donor X. For each direction, *N* = 1000 permutations were performed. In each permutation, a focal replicate (e.g. X_i_) was paired with a randomly sampled replicate from the opposing donor (e.g. Y_j_), with sampling conducted with replacement (i.e. a given replicate could be selected multiple times across permutations). This procedure generated a null distribution of compositional mixtures while maintaining independence across permutation iterations. Within each permutation, paired replicate abundances were summed at the OTU level. These summed profiles were averaged across the six biological replicates per donor and across all 1000 permutations to generate a stable mean directional mixture profile.

The directional mixtures were defined mathematically as follows:


(1) \begin{equation*} {Sim}_{X\to Y}^{(i)}=\frac{1}{N}\sum_{k=1}^N\left(\frac{1}{6}\sum_{j=1}^6\left({X}_i+{Y}_j^{(k)}\right)\right) \end{equation*}

(2) \begin{equation*} {Sim}_{Y\to X}^{(i)}=\frac{1}{N}\sum_{k=1}^N\left(\frac{1}{6}\sum_{j=1}^6\left({Y}_i+{X}_j^{(k)}\right)\right) \end{equation*}

The final simulated community profile was calculated as the mean of both directional mixtures:


(3) \begin{equation*} {Sim}_{final}^{(i)}=\frac{1}{2}\left({Sim}_{X\to Y}^{(i)}+{Sim}_{Y\to X}^{(i)}\right) \end{equation*}

This procedure was repeated for each of the six biological replicates per donor pair, resulting in six BANM-simulated coalescence profiles (taxon-by-sample abundance matrices). Each replicate therefore reflects bidirectional, randomized, and balanced donor contributions. BANM-generated communities were subsequently used in downstream ecological and network analyses.

### Balanced donor-sum simulation

To evaluate whether empirical MCC outcomes differed from expectations derived purely from direct donor blending, we implemented a BDS simulation. In this framework, donor communities were directly summed while enforcing equal donor contributions at the replicate level, thereby preserving donor-derived biotic structure without stochastic reshuffling of taxa. For each donor pair (X + Y; e.g. AxB, AxC, or BxC), six simulated mixtures were generated by randomly pairing one replicate from donor X with one replicate from donor Y, without replacement. For a given simulation set *s* and mixture replicate *r*, the simulated abundance of taxon *t* was defined as follows:


(4) \begin{equation*} {Sim}_{t,r}^{(s)}={x}_{t,{I}_{s,r}}^{(X)}+{x}_{t,{J}_{s,r}}^{(Y)} \end{equation*}

where ${x}_{t,{I}_{s,r}}^{(X)}$ and ${x}_{t,{J}_{s,r}}^{(Y)}$ represent the abundance of taxon *t* in replicate ${I}_{s,r}$ of donor X and replicate ${J}_{s,r}$ of donor Y, respectively. Indices ${I}_{s,r}$ and ${J}_{s,r}$ were drawn without replacement from the six biological replicates of donors X and Y. To account for stochastic variability in replicate pairing, this procedure was independently repeated 20 times per treatment combination (donor pair × dilution × time), generating 20 BDS simulation sets, each consisting of six simulated mixture replicates. These BDS-generated communities were subsequently used in downstream ecological and network analyses.

### Microbial co-occurrence network analysis

Microbial co-occurrence networks were constructed using the SparCC algorithm [31], implemented via FastSpar [32], a C++ implementation designed to estimate pairwise correlations from compositional data. Correlations were calculated using processed OTU count data across replicates for each donor, BANM and BDS simulations, and empirical treatment combinations (soil × biotic dilution × time). Statistical significance was assessed using 1000 permutation-based bootstrap iterations. Only statistically significant correlations (*P* < .01) with a magnitude (*ρ*) < −0.7 or > 0.7 were retained for network construction, consistent with thresholds commonly applied in microbial co-occurrence network studies [33, 34]. In the resulting networks, nodes represent OTUs displaying at least one significant correlation, and edges represent statistically significant pairwise correlations between OTUs. Network topological properties were calculated using the igraph package in R [35] and visualized in Gephi v0.10 [36] (see Supplementary Methods for details). The following metrics were quantified: network size (number of nodes), connectivity (number of edges), average degree (mean number of edges per node), average strength (mean weighted degree), positive-to-negative edge ratio, diameter, average clustering coefficient, density, relative modularity (RM), relative nestedness (RN), and positive and negative cohesion. Because a single network was inferred for each donor, BANM, BDS, and empirical treatment combination dataset, direct comparisons of network-level properties such as size, connectivity, and density were interpreted descriptively. Patterns of species co-occurrence and their topological organization (e.g. network size, connectivity, clustering, and modularity) are hereafter collectively referred to as network architecture.

To evaluate network structural stability following MCC, robustness, fragmentation, and vulnerability were assessed through node removal simulations (see Supplementary Methods). Network robustness was defined as the proportion of nodes remaining after either random (unweighted) or targeted (abundance-weighted) node removal [14, 19]. Robustness was calculated across twenty levels of node removal (5% to 100%), with 999 permutations per removal level. Changes in robustness as a function of node removal were modeled using linear regression, and the regression breakpoint was used to estimate the network resistance threshold to node loss. Network fragmentation was defined as the ratio of the number of disconnected components (*CL*) to the number of remaining nodes (*N*) under the same gradient node-removal framework [14]. Fragmentation trajectories were modeled using linear regression, and the slope was used to quantify the fragmentation rate, with higher values indicating faster structural disintegration following node removal. Network vulnerability was assessed based on changes in global network efficiency following removal of the most influential node [14], thereby quantifying network sensitivity to targeted perturbation [21]. Definitions of network topological metrics and stability simulations are provided in Supplementary Table S1.

To assess the sensitivity of network-level patterns to correlation threshold selection, we additionally reconstructed all empirical networks using |*ρ*| ≥ 0.6 and |*ρ*| ≥ 0.8, while maintaining *P* < .01, and compared the resulting network properties with those obtained using the |*ρ*| ≥ 0.7 threshold applied in the main analysis. Details of the correlation threshold sensitivity analysis are provided in the Supplementary Methods.

### Statistical analysis

All statistical analyses and visualizations were performed in R (v4.4.1). Changes in the number of significant nodes and edges between donor communities, BANM and/or BDS simulations, and empirical outcomes were tracked over time to assess network reassembly patterns. Proportional differences were compared using Monte Carlo Pearson’s chi-square tests (*N* = 10 000 permutations) or Fisher’s exact tests, as appropriate. Network topological parameters were categorized into two groups following Kajihara and Hynson [14]: (i) stability metrics, including network robustness (weighted and unweighted), fragmentation, and vulnerability; and (ii) complexity metrics, including network size, connectivity, average degree, density, clustering coefficient, relative nestedness (RN), relative modularity (RM), positive-to-negative edge ratio, and average positive and negative cohesion. Differences in network topological parameters among treatments were tested using analysis of variance (ANOVA) followed by Tukey’s HSD post hoc tests (α = 0.05), implemented in the agricolae package [37]. Linear mixed-effects models were used to evaluate variation in network complexity metrics across biotic dilution levels using the lmer function in the lme4 package [38]. Time was included as a fixed effect, and network identity (soil mixture × biotic dilution) was included as a random effect to account for non-independence among repeated measurements derived from the same network. Changes in stability metrics across the biotic dilution gradient were assessed using linear regression models implemented with the lm function in R. The influence of community diversity (observed OTUs and Shannon index) on network stability and complexity was evaluated using redundancy analysis (RDA) for each MCC treatment, implemented with the rda function in the vegan package [39]. Statistical significance of the constrained model was tested using permutation-based ANOVA with 999 permutations.

## Results

### Microbial community coalescence reorganizes association networks beyond null and biotic-blending expectations

**Figure 1:**
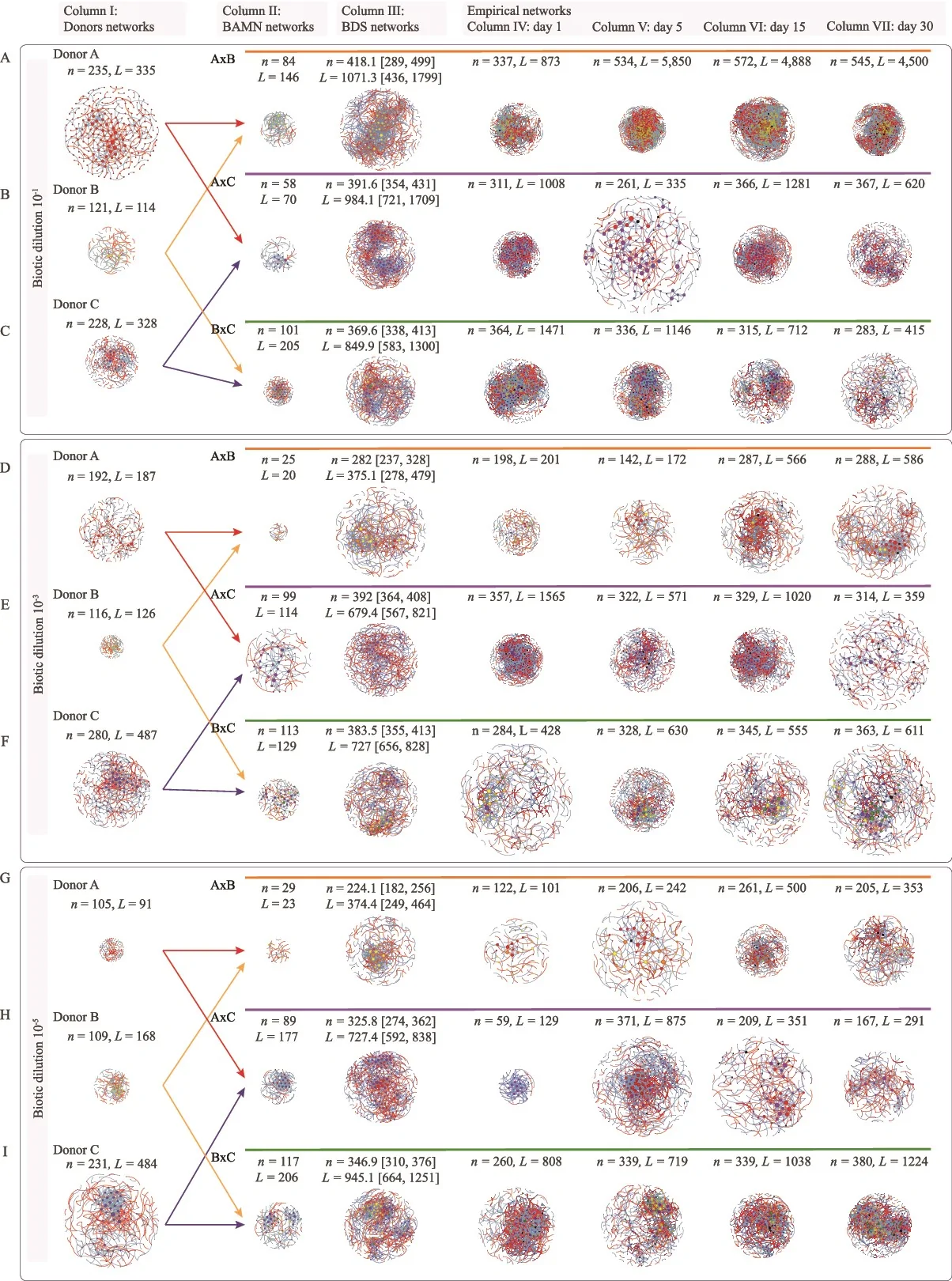
Microbial co-occurrence networks of donor communities (A, B, and C; Column I), BANM (Column II) and BDS (Column III) simulations, and empirical coalescence outcomes (AxB, AxC, and BxC) on Days 1 (Column IV), 5 (Column V), 15 (Column VI), and 30 (Column VII) following MCC across three biotic dilution levels (10^−1^, 10^−3^, and 10^−5^). Panels correspond to treatment and dilution levels as follows: AxB^−1^ (A), AxC^−1^ (B), BxC^−1^ (C), AxB^−3^ (D), AxC^−3^ (E), BxC^−3^ (F), AxB^−5^ (G), AxC^−5^ (H), and BxC^−5^ (I). Each node represents an OTU in the network, and node size is proportional to its number of connections (node degree). Network size denotes the total number of nodes (*n*), and connectivity refers to the total number of edges in the network (*L*). For BDS simulations, values represent the mean across twenty simulations, and the range in brackets indicates the minimum and maximum values of *n* and *L* across the twenty simulations. Edges represent significant SparCC correlations (*ρ* < − 0.7 in red and *ρ* > 0.7 in blue; *P* < .01), with blue edges indicating positive correlations and red edges indicating negative correlations. Network visualizations were generated using the Fruchterman–Reingold layout algorithm. The spatial extent of each network was normalized across all networks and does not represent ecological distances, but is rather meant to facilitate visualization.

Co-occurrence networks of donor communities, empirical coalescence outcomes, and BANM and BDS simulations were constructed based on strong and significant SparCC correlations (*ρ* > 0.7 or < −0.7, *P* < .01; Fig. 1; Supplementary Table S2). Visual inspection of networks suggests empirical networks frequently diverge from both BANM and BDS expectations, thus motivating the quantitative assessments presented below. Across treatments and time points, empirical MCC networks generally exhibited larger network size (i.e. number of significant nodes) than both donor networks and BANM simulations. Exceptions were observed for AxB^−3^ on Days 1 and 5, and AxC^−5^ on Days 1, 15, and 30. In contrast, empirical network size varied relative to BDS expectations, with values exceeding, falling below, or remaining within the BDS simulation range depending on treatment and time. Empirical networks were larger than BDS simulations in AxB^−1^ (Days 5, 15, and 30), AxB^−5^ (Day 5), and BxC^−5^ (Day 30). Smaller empirical networks were more frequently observed in AxB^−3^ (Days 1 and 5), AxB^−5^ (Day 1), AxC^−1^ (Days 1 and 5), AxC^−3^ and AxC^−5^ (Days 1, 15, and 30), BxC^−1^ (Days 5, 15, and 30), BxC^−3^ (Days 1, 5, and 15), and BxC^−5^ (Day 1). In the remaining time points, empirical network sizes fell within the BDS range. Together, these patterns indicate that stochastic reshuffling alone (BANM) fails to reproduce the observed association network size following MCC, while direct biotic blending (BDS) only partially captures the empirical variation in network architecture.

**Figure 2:**
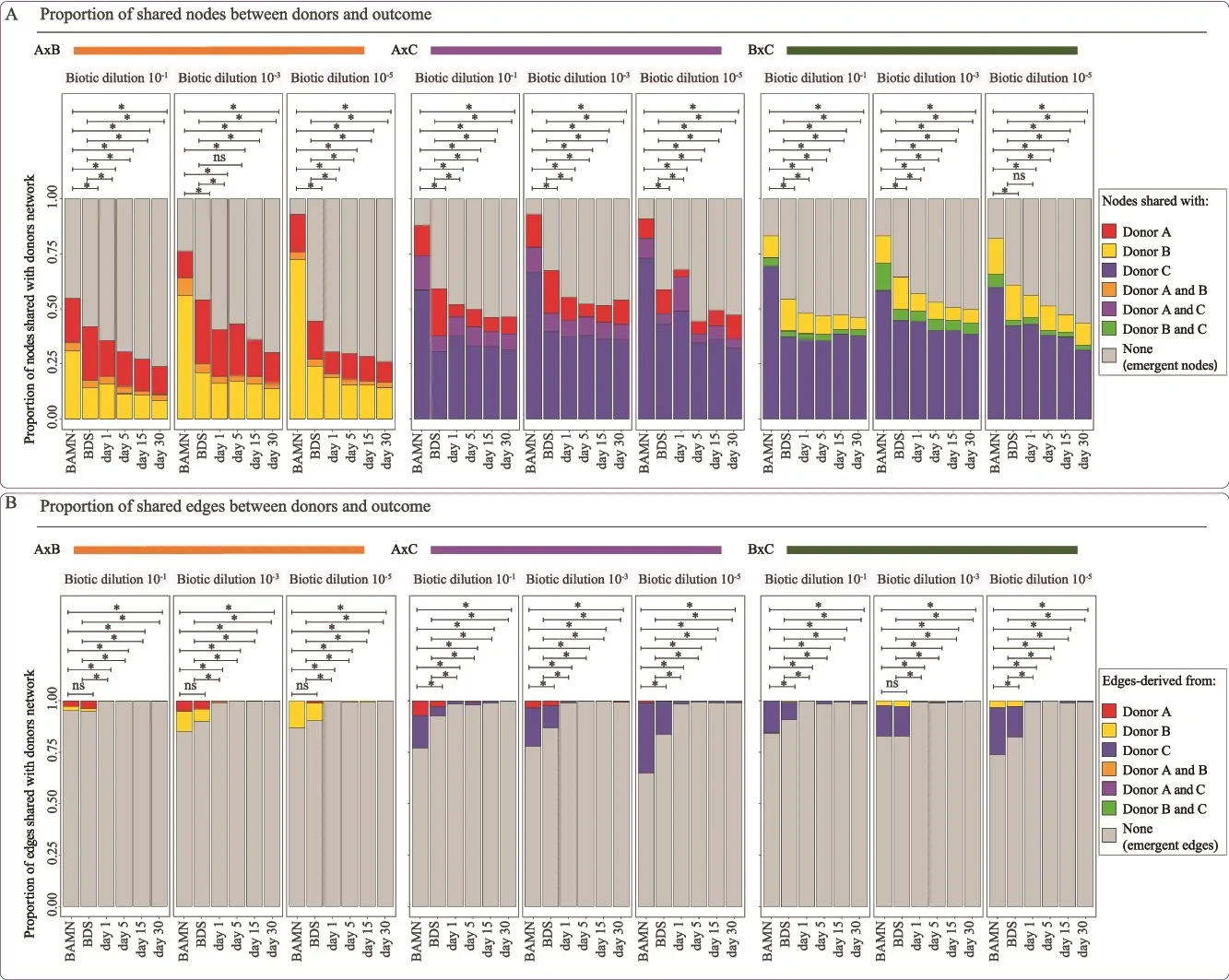
Proportions of shared nodes (A) and edges (B) between donor networks and BANM simulations, BDS simulations, and empirical coalescence outcomes. Significant differences between the proportions expected under the simulation models and those observed in empirical outcomes are indicated by “*” (*P* < .05). Statistical comparisons were performed using Pearson’s chi-square tests with Monte Carlo permutations for shared node distributions and Fisher’s exact tests for donor-derived edge distributions.

Analysis of donor-derived node persistence further revealed substantial reorganization of the co-variation structure following MCC (Fig. 2A; Supplementary Table S3). The proportions of donor nodes retained in empirical outcome networks differed significantly from those expected under BANM and BDS simulations in nearly all cases (Monte Carlo Pearson’s Chi-square test, *P* < .05), except for AxB^−3^ on Day 5 and BxC^−5^ on Day 1. BANM simulations predicted high persistence of donor nodes (>54% for AxB, >87.9% for AxC, and > 82% for BxC), whereas BDS simulations yielded intermediate expectations (34%–61.6% for AxB, 53.7%–72.3% for AxC, and 50%–71.3% for BxC). In contrast, empirical MCC networks exhibited consistently lower donor-node persistence, ranging from 32.2% to 76.1%, with exceptions for AxB^−1^ and AxC^−5^ on Day 1 matching BDS simulation expectations (Supplementary Table S3). These results indicate extensive turnover of taxa participating in significant associations, consistent with pronounced change in the correlation structure after MCC. A similar pattern was observed for donor-derived edges (Fig. 2B; Supplementary Table S4). The proportion of persistent donor correlations did not differ significantly between BANM and BDS simulations for AxB and BxC^−3^ (Fisher’s exact test, *P* > .05), but both simulation frameworks differed significantly from empirical networks (Fisher’s exact test, *P* < .01). Notably, empirical MCC networks exhibited >97.9% novel correlations, far exceeding the range predicted by BANM and BDS simulations, with no donor modules found to be conserved (Supplementary Table S4). Collectively, these results demonstrate that MCC results in an extensive reorganization of association networks that cannot be explained by stochastic reshuffling of abundances (BANM) or by simple compositional blending of donor communities (BDS).

### Coalescence modifies network architecture and compartmentalization relative to donors and reference models

**Figure 3:**
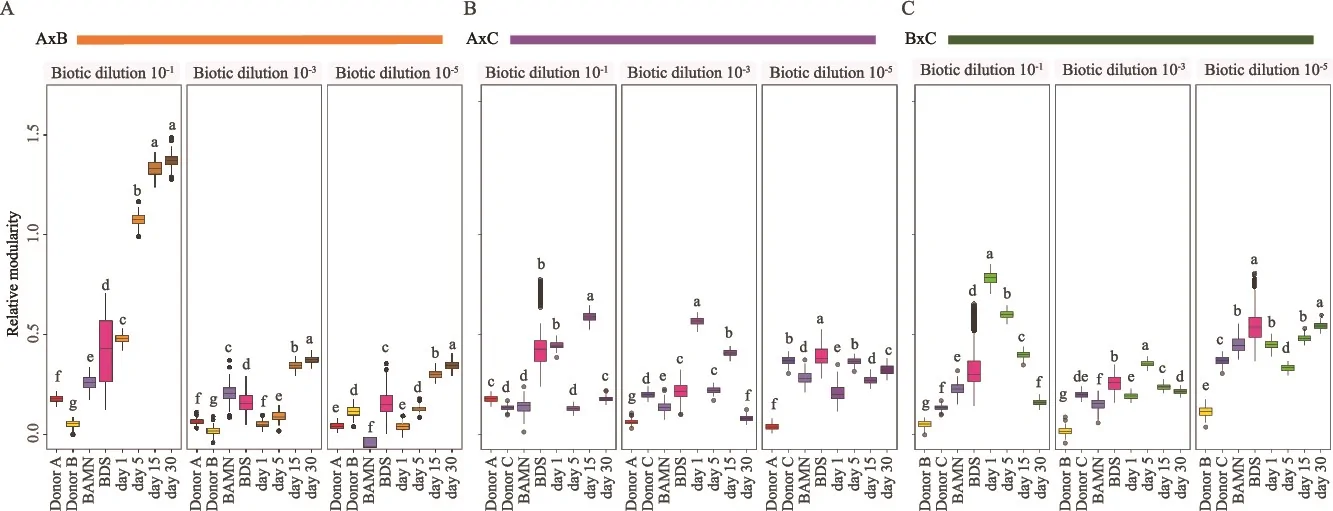
Boxplots of network relative modularity (RM) for AxB (A), AxC (B), and BxC (C) treatments, including donor networks, BANM and BDS simulations, and empirical coalescence outcomes across three biotic dilution levels (10^−1^, 10^−3^, and 10^−5^) and sampling times (Days 1, 5, 15, and 30). Different letters indicate statistically significant differences among treatments based on Tukey’s HSD test (*P* < .05).

To determine whether MCC alters higher-order features of the correlation structure beyond simple node and edge turnover, we compared network architectural metrics [including size, connectivity, average degree, clustering coefficient, cohesion, relative modularity (RM), and relative nestedness (RN)] across donors, BANM, BDS simulations, and empirical outcomes (Supplementary Table S2). Relative modularity (RM), used here as an index of compartmentalization within the association network, differed consistently among simulated models and empirical outcomes (Fig. 3). BANM simulations frequently produced RM values that were similar to or higher than those observed in donor networks (HSD Tukey test, *P* < .05), with exceptions for AxB^−5^ and BxC^−3^ (*P* > .05). In contrast, BDS simulations consistently predicted higher RM than the donors across treatments (HSD Tukey test, *P* < .05), suggesting that direct compositional mixing alone tends to increase compartmentalization of the correlation structure relative to the original donor networks.

Empirical MCC networks exhibited RM values that were generally higher than those expected under stochastic reshuffling (BANM), indicating that coalescence does not simply dilute or randomize association structure. In many cases, empirical RM values were within the range predicted by BDS simulations, consistent with partial preservation of compositional influences on compartmentalization. However, several treatment–time combinations deviated from BDS expectations. RM exceeded the BDS range in AxB^−1^ (Days 5 to 30), AxB^−3^ (Days 15 and 30), and AxC^−3^ and BxC^−1^ (Day 1), whereas RM fell below BDS expectations in AxC^−1^ (Days 5 and 15), AxC^−3^ (Day 30), AxC^−5^ (Day 1), and BxC^−5^ (Day 5) (HSD Tukey test, *P* < .05). These deviations suggest that empirical coalescence outcomes cannot be fully explained by either stochastic redistribution of abundances or additive mixing of donor communities. Across additional architectural metrics (degree, clustering coefficient, cohesion, and connectivity; Supplementary Table S2), empirical networks frequently diverged from both reference models, further supporting the conclusion that MCC contributes to dynamic patterns of taxon co-variation. Notably, shifts in clustering coefficient and cohesion suggest reorganization of outcome association density and balance between positive and negative correlations, reinforcing that coalescence modifies not only the size of the network but also its internal structural arrangement. Together, these results demonstrate that MCC induces measurable changes in network architecture and compartmentalization relative to both donor configurations and reference expectations.

### Biotic dilution modulates association complexity and structural stability in a treatment-specific manner

**Figure 4:**
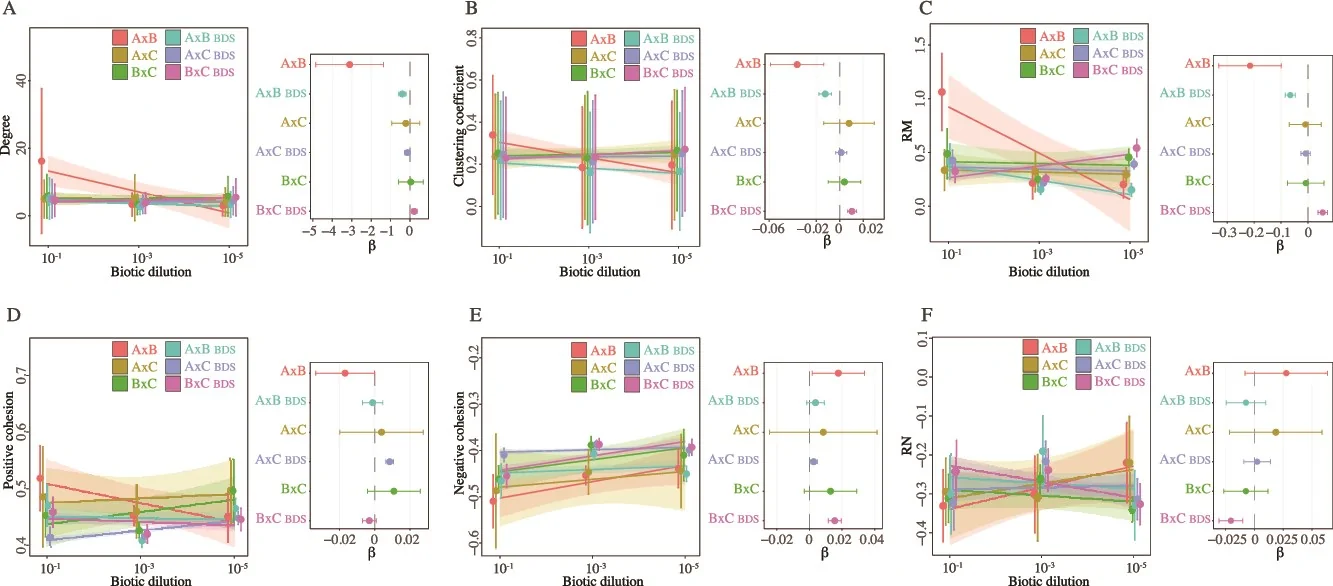
Relationships between network complexity metrics and biotic dilution levels, including degree (A), clustering coefficient (B), relative modularity (C), positive cohesion (D), negative cohesion (E), and relative nestedness (F). Points represent mean values (± SD), and solid lines indicate fitted trends from linear mixed-effects models with 95% confidence intervals. Colors denote MCC treatments (AxB, AxC, BxC) and their corresponding BDS simulations.

**Figure 5:**
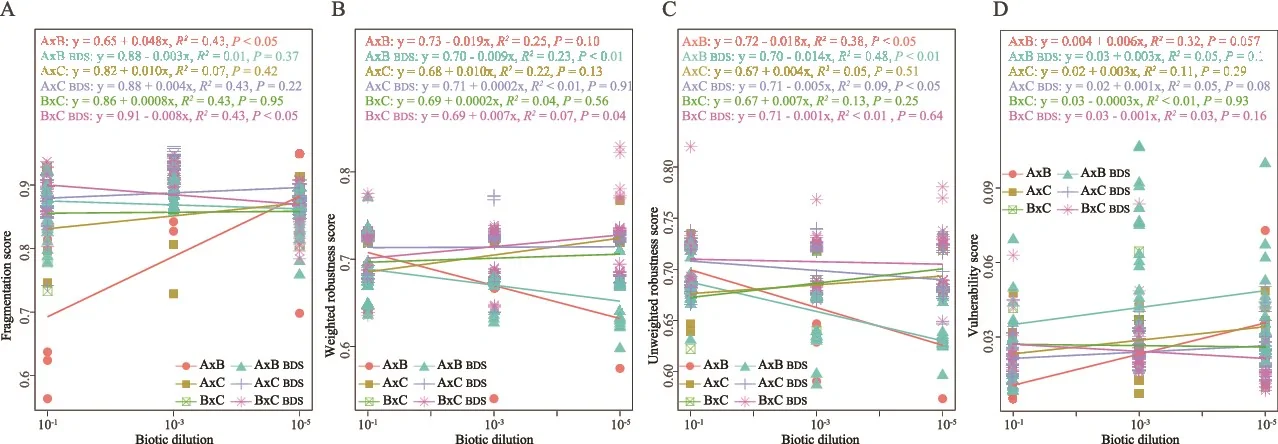
Relationships between network stability metrics and biotic dilution levels, including fragmentation (A), weighted robustness (B), unweighted robustness (C), and vulnerability (D). Points represent network observations at each sampling time. Solid lines indicate fitted linear regression models for each treatment, and the corresponding regression equations are shown within the panels.

To assess whether the intensity of biotic dilution influences the emergent correlation structure following MCC, we examined how network complexity and structural stability metrics varied across dilution gradients within each treatment (Figs 4 and 5; Supplementary Table S2 and S5 and Figs S1–S4). Across treatments, dilution-dependent patterns were predominantly restricted to the AxB, whereas AxC and BxC showed limited or inconsistent responses. In the empirical AxB networks, linear mixed-effects models revealed that increasing biotic dilution was associated with significant declines in multiple architectural complexity metrics, including average degree, clustering coefficient, relative modularity (RM), and positive cohesion (*β* < 0; 95% CI; Fig. 4). In contrast, negative cohesion increased along the dilution gradient (*β* > 0; 95% CI), indicating a shift in the balance of positive and negative associations as community biotic dilution is reduced. These dilution-associated patterns in complexity were partially supported by BDS simulations for degree, clustering coefficient, and RM (*β* < 0; 95% CI), suggesting that some metrics of architectural sensitivity to dilution can be explained by compositional blending, but not entirely.

Dilution effects also extended to measures of structural stability of the correlation networks (Fig. 5). In empirical AxB networks, network fragmentation increased significantly with biotic dilution (linear model, *R^2^* = 0.43, *P* < .05), indicating a higher propensity for the association network to fragment into disconnected subsets under node removal as dilution intensified. Network vulnerability exhibited a marginally significant positive relationship with dilution (*R^2^* = 0.32, *P* = .057), suggesting increased sensitivity of the correlation structure to targeted perturbation. Consistently, unweighted robustness declined significantly along the dilution gradient (*R^2^* = 0.38, *P* < .05), reflecting reduced structural resistance of the association network to random node loss at higher dilution levels. Among these stability metrics, concordant BDS patterns were observed only for unweighted robustness (*R^2^* = 0.48, *P* < .001), indicating that dilution-induced reductions in robustness are partly captured by direct taxa mixing, whereas changes in fragmentation and vulnerability reflect emergent structural reorganization beyond compositional effects. In contrast, AxC and BxC treatments did not exhibit consistent or significant dilution-dependent trends in either complexity or stability metrics (Figs 4 and 5), supporting that the impact of dilution on network architecture is system-specific.

### Community diversity predicts network architecture only in specific coalescence contexts

**Figure 6:**
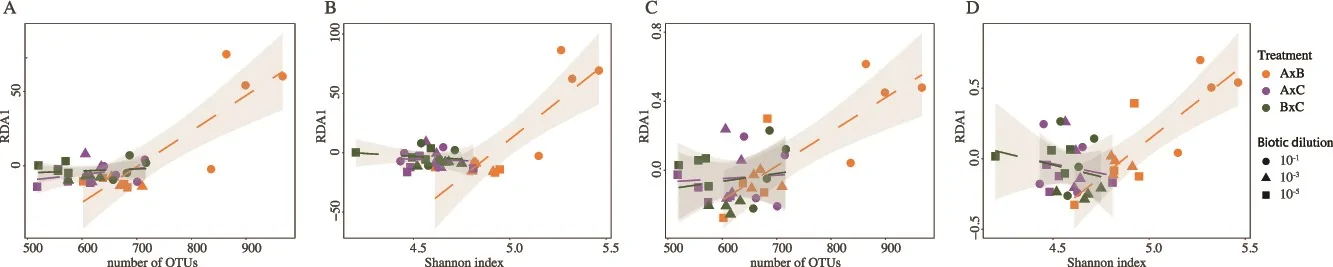
Relationships between community diversity and multivariate network architecture. RDA was used to evaluate how variation in network topological properties was associated with community diversity metrics. For each treatment, network architecture was summarized by the first constrained ordination axis (RDA1) from separate RDAs performed for network complexity metrics (A, B) and network stability metrics (C, D). Panels show associations between RDA1 scores and observed OTU richness (A, C) and Shannon diversity (B, D). Points represent individual networks and are colored by MCC treatment (AxB, AxC, BxC) and shaped according to biotic dilution level. Dashed lines indicate fitted linear regressions with 95% confidence intervals. Permutation-based ANOVA (999 permutations) revealed significant relationships between diversity and both network complexity and network stability in AxB networks (adjusted *R^2^* > 0.5, *P* < .01), whereas no significant relationships were detected for AxC or BxC networks (*P* > .05; see Supplementary Table S6).

To evaluate whether variation in outcome community diversity explains differences in emergent network architecture (e.g. network size and connectivity) and structural stability (e.g. fragmentation and robustness) following MCC, we performed redundancy analyses (RDA) linking alpha-diversity metrics (observed OTU richness and Shannon index) to network complexity and stability parameters (Fig. 6; Supplementary Table S6). In the empirical AxB treatment, community diversity was a strong predictor of network architecture. Observed OTU richness explained 73.8% of the total variance in network complexity (Adj *R^2^* = 0.712, *F* = 28.16, *P* = .003, 999 permutations) and 56.1% of the variance in network stability (Adj *R^2^* = 0.517, *F* = 12.78, *P* = .005, 999 permutations). Similarly, Shannon diversity accounted for 72.2% of the variance in network complexity (Adj *R^2^* = 0.694, *F* = 25.95, *P* = .005, 999 permutations) and 57.3% of the variance in network stability (Adj *R^2^* = 0.5300, *F* = 13.41, *P* = .001, 999 permutations). These results suggest a strong coupling between alpha-diversity and the correlation structure in AxB, such that increases in taxonomic diversity were associated with coordinated shifts in both architectural complexity and structural robustness of the association network. In contrast, no significant relationships between alpha-diversity metrics and network complexity or stability were detected in AxC or BxC empirical treatments (*P* > .05). Despite comparable ranges of diversity across biotic dilution treatments (Supplementary Figs S1 and S2), diversity failed to explain variation in the correlation structure in these contexts. Together, these findings demonstrate that diversity–architecture coupling is not a universal outcome of MCC but instead emerges in a system-specific fashion. In AxB, taxonomic diversity strongly predicts the configuration and resilience of the association network, whereas in AxC and BxC, network architecture appears decoupled from alpha-diversity. This context dependence suggests that the influence of diversity on emergent co-variation structure is contingent on biotic and abiotic properties in the donors and coalescence regime rather than being an inherent property of diversity alone.

## Discussion

### Microbial community coalescence restructures association architecture beyond stochastic and additive expectations

Species co-occurrence networks provide descriptive representations of how taxa are organized within communities, capturing patterns of positive and negative co-variation that may reflect the combined influence of biotic interactions and environmental filtering on community assembly. Accordingly, our interpretations focus on changes in the structure of association networks rather than direct inference of ecological interactions and processes. In this study, we examined soil microbial community reassembly following MCC through two complementary structural dimensions: (i) donor contribution and node persistence within outcome networks, and (ii) the persistence and redistribution of donor-derived correlations (edges) after coalescence. By benchmarking empirical networks against a stochastic null model (BANM) and a donor-preserving compositional blending model (BDS), we tested whether post-coalescence network architectures deviate from expectations based on neutral reshuffling or additive taxa mixing.

Across treatments, empirical networks showed pervasive structural rearrangement. The emergence of new significant nodes exceeded both BANM and BDS expectations, and empirical networks frequently displayed larger sizes than stochastic predictions (Figs 1 and 2A). Because nodes represent taxa displaying strong correlations, their inclusion beyond that of null expectations suggests that neutral processes do not fully explain MCC reassembly [21, 40]. Instead, our observations are consistent with deterministic ecological processes, including environmental filtering and biotic interactions, contributing to community reassembly following MCC, although the relative importance of these processes cannot be inferred directly from the network analyses. However, similar expansions in network size under environmental perturbation (e.g. climate warming) have been interpreted as a response to abiotic change restructuring patterns of species association [21]. Accordingly, in the context of MCC, it has been conceptualized that simultaneous mixing of biotic assemblages and abiotic matrices modifies resource availability, species competitive interactions, and habitat filters [5], and, in specific cases, this may allow previously rare taxa to become prominent within the association network [41, 42]. Such dynamics closely parallel invasion ecology, where successful colonizers establish under altered competitive and environmental contexts [43, 44], and taxa previously dominating in the donor context can be replaced by emergent species configurations [45].

Moreover, we found our empirical networks to exhibit >97% novel correlations and negligible donor-module persistence, far exceeding both BANM and BDS predictions (Fig. 2B). This extensive reorganization of donors’ species associations is expected when communities are mixed, resulting in variations in species relative proportions and new ecological conditions during MCC [46]. Whereas BANM captures stochastic mixing and BDS preserves donor co-variation structure, neither of these simulations reproduced the magnitude of restructuring in empirical species co-occurrence structure. This divergence between null (BANM), additive (BDS), and empirical outcomes supports the conceptualization that MCC results in a dynamic interplay of biotic and abiotic selective pressures [15], via changes in resource availability and competitive species interactions [43, 44]. Together, these patterns are consistent with MCC reorganizing correlation architecture among taxa through a dynamic interplay of biotic and abiotic filters, including feedback between species interactions and environmental constraints, aligning with theory and other experimental observations [5, 7]. With that, the outcome network architecture deviates substantially from a simple compositional mixing outcome as BDS predicts. Importantly, higher-order architectural metrics further support this conclusion. For instance, our empirical networks frequently exhibited higher RM than stochastic expectations and, in some cases, deviated from additive blending predictions (Fig. 3). Increased modularity implies stronger compartmentalization, often interpreted as reflecting niche differentiation or shared responses to environmental variations [11, 14, 47]. From a community assembly perspective, MCC may expand species niche breadth in the outcome community by combining taxa with distinct metabolisms, intensifying selection, and promoting niche segregation [48]. Therefore, the resulting compartmentalization is consistent with emergent ecological organization following mixing rather than simple inheritance of donor-derived co-occurrence structure.

### Context-dependent stability regimes and diversity–network architecture coupling across coalescence outcomes

While MCC consistently restructured taxa association architecture, its consequences for network complexity and structural stability were strongly context dependent. For instance, we found biotic dilution to influence complexity and stability primarily in AxB treatments, whereas AxC and BxC mixtures exhibited limited responses (Figs 4 and 5). In AxB, increasing biotic dilution reduced network complexity (i.e. degree, clustering coefficient, RM) and robustness (Fig. 4A–C and Fig. 5C), while increasing fragmentation and vulnerability (Fig. 5A and D). In our experimental design, the biotic dilution approach was intentionally used to randomly disrupt ecological interactions among species and weaken community cohesiveness (see [49]). Consequently, lower dilution levels are expected to preserve tighter patterns of species interactions and co-occurrence, which may favor taxa engraftment by maintaining or rearranging species correlations rather than promoting taxa loss following MCC. In contrast, treatments involving donor C [7] exhibited architectural configurations that were less sensitive to biotic dilution. In a previous study, we demonstrated that stringent abiotic filtering modulated community reassembly in treatments mixed with donor C (i.e. AxC and BxC treatments), and this filtering was largely regulated by shifts in soil pH and nitrogen pools (see [7]). Thus, although the number of donor communities examined here is limited, these patterns suggest that the relative strength of environmental filtering may modulate how biotic perturbations affect structural network outcomes. Importantly, these network-level patterns were generally robust to the correlation threshold used for network construction, with most network properties showing strong consistency across correlation cutoffs of |*ρ*| ≥ 0.6 to |*ρ*| ≥ 0.8 (Supplementary Results and Supplementary Fig. S5). Network robustness estimates, however, were comparatively more sensitive to threshold selection, particularly for BxC under the most stringent cutoff (|*ρ*| ≥ 0.8), highlighting that stability simulations derived from threshold-dependent network reconstruction should be interpreted with caution.

Patterns linking community diversity to network architecture (i.e. patterns of species co-occurrence) were also contingent on coalescence context. Notably, in AxB treatments, alpha diversity explained a substantial proportion of the variation in both network complexity and stability metrics. Whereas in AxC and BxC mixtures, diversity did not predict network architecture despite comparable diversity gradients (Fig. 6). Although diversity has frequently been associated with resilience, coexistence, and emergent stability [50–52], our findings suggest that its influence on structural organization, here co-occurrence network properties, becomes apparent primarily under biotically driven assembly regimes, for example, in our treatment AxB. On the other hand, when abiotic filtering exerts a stronger influence over reassembly trajectories (i.e. mixtures with donor C [7]), similar variations in diversity may not proportionally restructure correlation networks (see Supplementary Figs S1 and S2) [53]. As such, while BDS simulations partially captured compositional mixing effects, they did not reproduce these context-dependent diversity–architecture relationships. This indicates that emergent structural organization, such as stability, complexity, and stable coexistence, may depend on a combination of ecological properties (e.g. species presence and interactions, community cohesiveness, and responses to environmental conditions) rather than diversity alone. Although our analyses focused on emergent community-level network architecture, identifying the specific taxa or microbial groups contributing to these emergent association patterns represents an important direction for future research. Combining network analyses with targeted experimental validation will be necessary to determine the ecological roles of these taxa during MCC.

Collectively, our findings indicate that MCC generates regime-specific patterns of network complexity and stability modulated by the relative dominance of biotic interactions and environmental filtering. Here, MCC not only restructures species association patterns but also influences the architectural complexity of species co-occurrence networks. In summary, our results support the conceptualization that MCC is a dynamic ecological process that reorganizes correlation architecture and the structural stability of the inferred association network through context-dependent ecological feedbacks. Future work linking these structural shifts to ecosystem functioning will be critical to understanding the broader ecological consequences of MCC across natural and managed systems.

## Supplementary Material

Supplementary_information_Bresciani_et_al_2026_R3_ycag260

## Data Availability

The raw sequencing reads generated in this study have been deposited in the NCBI Sequence Read Archive (SRA) under BioProject accession PRJNA1147197. The code used to implement the BANM and BDS reference frameworks, along with example input files and documentation, is publicly available at: https://github.com/luanabresciani/coalescence-reference-frameworks.
