# Supplementary material for: Microbial community coalescence restructures co-occurrence architecture and generates regime-specific patterns of network complexity and stability
Source: ISME Commun. 2026 Sep 12;6(1):ycag260. doi: 10.1093/ismeco/ycag260 (PMC13615532; doi:10.1093/ismeco/ycag260)
Supplement: Supplementary file 1 [file supplementary_information_bresciani_et_al_2026_r3_ycag260.docx]

**SUPPLEMENTARY INFORMATION**

**Microbial community coalescence restructures co-occurrence architecture and generates regime-specific patterns of network complexity and stability**

**Short Title: Microbial co-occurrence following coalescence**

**Authors**

Luana Bresciani^1,2^, Gordon F Custer^1,2,3^, Francisco Dini-Andreote^1,2*^

**Affiliations**

^1^Department of Plant Science & Huck Institutes of the Life Sciences, The Pennsylvania State University, University Park, PA, USA

^2^The One Health Microbiome Center, Huck Institutes of the Life Sciences, The Pennsylvania State University, University Park, PA, USA

^3^Department of Natural Sciences, School of Agriculture and Natural Resources, The University of Maryland Eastern Shore, Princess Anne, MD, USA

***Corresponding author:** Francisco Dini-Andreote, Department of Plant Science & Huck Institutes of the Life Sciences, The Pennsylvania State University, University Park, State College, PA, 16802, USA. E-mail: andreote@psu.edu, phone: +1 814 8632188.

**Glossary of Network Topological Metrics**

This supplementary **Table S1** provides detailed definitions of all network topological metrics calculated in this study, including nodes, edges, degree, clustering coefficient, density (or connectance), size, connectivity, average degree, average cohesion, positive-to-negative edge ratio, relative modularity, relative nestedness, vulnerability, robustness, and fragmentation indices.

**Supplementary Methods**

*Microbial co-occurrence network construction and characterization*

Network density, which measures the proportion of realized edges among all possible associations, was calculated by dividing the number of observed edges (*L*) by the maximum number of potential edges, as follows:

$C=L/{(\frac{N\left( N-1 \right)}{2})}$ **[S1]**

, where *N* is the number of nodes in the network [1]. This metric indicates the degree of network density relative to network size.

Network relative modularity (RM), which measures the degree of compartmentalization of a network into different modules compared with random expectation, was calculated as [2]:

$RM=\left( M-\overline{M_{r}} \right)/{\overline{M_{r}}}$ **[S2]**

, where *M* is the modularity of each constructed network, calculated using the Louvain modularity detection algorithm implemented in the cluster_louvain function of the “igraph” R package (v.2.1.4) [3], and$\overline{M}_{r}$ is the average modularity computed from 100 randomized networks generated for each constructed network following the Maslov-Sneppen procedure, which preserves node degree [4].

Network relative nestedness (RN) measures the degree of hierarchical organization in the co-occurrence correlation network through the tendency for nodes with few significant correlations to correlate with a subset of partners of nodes with many correlations [5]. Similarly to RM, RN was calculated relative to random expectation, as follows:

$RN=\left( N-\overline{N_{r}} \right)/{\overline{N_{r}}}$ **[S3]**

, where *N* is the nestedness of each constructed network assessed using unweighted graph (i.e., binary correlation matrix) and NODF algorithm (‘nested’ function in “bipartite” package in R) [6], and $\overline{N}_{r}$ is the nestedness score calculated from the average of 100 randomized networks generated using the r2dtable function (in the “bipartite” package in R) [6].

Network cohesion, which quantifies the degree of network connectivity, was calculated using an abundance-weighted and null model–corrected approach [7]. Pairwise correlations were calculated from taxon-by-sample abundance matrix, followed by null model correction based on 999 null model iterations. Positive and negative cohesion were calculated independently using positive and negative correlations, respectively, as follows:

$cohesion= \sum_{i=1}^{m} {abundance}_{i} x {connectedness}_{i}$ **[S4]**

, where *m* is the total number of taxa in a community. To ensure consistency between sample-level cohesion metrics and all other network analyses, the dataset used for cohesion calculations followed the approach of Yuan et al. [2] and restricted the taxa to those significantly retained in the co-occurrence network (i.e., nodes). Pairwise correlations were computed from the relative abundance matrix and corrected using a taxa-shuffle null model [7].

Network robustness, which measures node persistence in the network after random and progressive node removal (e.g., species loss), was simulated using abundance-weighted and unweighted approaches [8, 9]. In both cases, nodes were randomly removed from the network along with their respective edges, and the remaining node mean interaction strength (MIS) was assessed based on the sum of its correlations with all other nodes, as follows:

${MIS}_{i}= \sum_{j\neq i} s_{ij} x b_{j}$ **[S5]**

, where *i* is the remaining node, *sij* is the correlation strength between nodes *i* and *j* (here SparCC-weighted correlations), and *b_j_* is the relative abundance of node *j*. For the unweighted approach, *b_j_* was set to 1 for all nodes to remove the influence of abundance. Nodes resulting in MIS less than or equal to zero were considered extinct or isolated and were therefore removed from the network, and the proportion remaining was reported as the network robustness. Simulations were performed for twenty levels of node removal (i.e., from 5 to 100%) with 999 permutations per level. For each treatment, the relationship between network robustness and the proportion of nodes removed was modeled through linear regression, and its breakpoint was identified using the “segmented” R package [10]. The regression breakpoint represents the point at which robustness changes along the gradient of node removal and assesses the network resistance threshold to species loss.

Network fragmentation was also assessed under progressive random node removal using simulation-based analysis [8]. Fragmentation was calculated as the ratio of the number of disconnected components (*CL*) to the number of remaining nodes (*N*) after node removal, as follows:

$f=CL/N$ **[S6]**

, where higher values indicate a more fragmented network structure. The simulation was bootstrapped 1,000 times for each network graph, averaged, and nodes were progressively removed from 0 to 100% to compute the fragmentation trajectory. The fragmentation rate was modeled using a linear regression model, and the slope was calculated using the coef function in the “stats” package in R [11].

Network vulnerability was assessed based on network global efficiency following node removal [8]. Global efficiency quantifies how rapidly information, or ecological effects, propagate across the network, using the average of the inverse shortest path length between all pairs of nodes, as follows:

$E=\frac{1}{n\left( n-1 \right)}\sum_{i\neq j} \frac{1}{d\left( i,j \right)}$ **[S7]**

, where *d(i,j)* is the number of edges in the shortest path length between nodes *i* and *j*, and *n* is the total number of nodes. Each individual node vulnerability was calculated as the relative decrease in global efficiency:

$V_{i}=(E-E_{i})/E$ **[S8]**

, where *E* is the global efficiency and *E_i_* is the global efficiency following the removal of node *i* and its edges [12]. Network vulnerability was computed as the maximum node vulnerability, representing the network sensitivity to the loss of its most critical node [2].

*Statistical analysis*

Differences between donors and outcome community structures were visualized using principal coordinate analysis (PCoA) based on Bray-Curtis dissimilarity and statistically tested via permutational multivariate analysis of variance (PERMANOVA) with 999 permutations using the adonis function in the “vegan” R package [13]. Community diversity metrics (i.e., observed OTU richness and Shannon index) were calculated using the estimate_richness function in the “phyloseq” R package [14], and differences were determined using ANOVA with Tukey’s HSD test (α = 0.05) via the “agricolae” R package [15]. Outcome taxonomy was assigned to its donors’ source (e.g., Soil A, Soil B, or both A and B) based on Venn Diagram overlap using the get_vennlist function in the “MicrobiomeProcess” R package [16]. This was used to characterize empirical, BAMN, and BDS coalescence communities and network graph profiles based on their taxa source. Statistical inference of network robustness and fragmentation was assessed using the robustness breakpoint and fragmentation slope, with 95% confidence intervals around the mean estimates.

*Network correlation threshold sensitivity analysis*

To evaluate the robustness of network-level patterns to correlation threshold selection, all empirical networks were reconstructed using three correlation magnitude cutoffs: |*ρ*| ≥ 0.6, |*ρ*| ≥ 0.7, and |*ρ*| ≥ 0.8, while maintaining a statistical significance criterion of *P* < 0.01. The |*ρ*| ≥ 0.7 cutoff corresponded to the threshold used in the main analyses. For each threshold, the same set of network properties was recalculated, including the number of nodes and edges, mean degree, density, clustering coefficient, ratio of positive to negative associations, relative modularity, relative nestedness, positive and negative cohesion, fragmentation, vulnerability, and weighted and unweighted robustness. Sensitivity to threshold selection was quantified by comparing the temporal trajectories of each network property obtained at |*ρ*| ≥ 0.6 and |*ρ*| ≥ 0.8 with those obtained using the original |*ρ*| ≥ 0.7 threshold. Spearman rank correlations were calculated separately for each network property and coalescence treatment across the 12 matched networks defined by dilution level and time point using the “cor.test” function in R.

**Supplementary Results**

*Community diversity and structure after MCC*

Changes in community alpha diversity after MCC were estimated using observed number of OTUs and Shannon index (**Figure S1 and S2**). In brief, the AxB and AxC outcomes tended to present a statistically similar number of OTUs and Shannon index values to one or both donors or values intermediate between them (Tukeys’s HSD, *P* > 0.05). In contrast, BxC outcomes showed a tendency toward a statistically higher number of observed OTUs compared to the donors, while the Shannon index remained similar. Across biotic dilutions, outcome communities showed a statistically higher number of OTUs at lowest biotic dilution level (i.e., 10^-1^) compared to intermediate (i.e., 10^-3^) and higher dilutions (i.e., 10^-5^) (Tukeys’s HSD, *P* > 0.05). This pattern was only observed for the Shannon index in AxB, whereas AxC and BxC treatments showed similar diversity values across dilution levels (**Figure S1 and S2**).

*Community network complexity and stability after MCC*

To determine how MCC affects outcome network complexity, changes in network size (i.e., number of nodes), connectivity (i.e., number of edges), clustering coefficient, node average degree, positive and negative cohesion, and relative modularity (RM) and nestedness (RN) were compared with the donors, BANM, and BDS simulation networks (**Table S2**). The number of nodes in the donor networks ranged from 105 to 280, and the number of edges ranged from 91 to 487 across dilution levels. In the BANM outcome networks, these parameters ranged between 25 and 117 for the number of nodes and 20 to 206 for the number of edges, whereas BDS simulations ranged between 224.1 and 418.1 nodes on average and between 374.4 and 1,071.3 edges on average. In empirical outcome networks, the number of nodes ranged from 59 to 572, and the number of edges ranged from 101 to 5,850. In brief, BANM networks tended to present statistically similar average node degree and clustering coefficients compared with one or both donors (Tukey’s HSD test, *P* < 0.05), whereas BDS simulations showed higher node degree and clustering coefficient in treatments AxC^-1^ and BxC^-1^, and higher node degree only in treatment BxC^-5^. However, in empirical networks, lower dilution treatments (i.e., 10^-1^) tended to present higher average node degree and clustering coefficient compared with the donors and both simulations (**Table S2**).

In network cohesion, BANM networks tended to present statistically similar, intermediate, or higher positive and negative cohesion, whereas BDS simulations showed statistically similar, intermediate, or lower cohesion values compared with the donors (**Table S2**). These results indicate opposing trends in the potential strengthening of cooperative and competitive interactions or filtering processes after MCC. However, empirical networks did not follow BANM and BDS simulation results and showed different trends across mixtures and biotic dilution treatments. Particularly, for empirical treatments AxB, statistically higher cohesion was observed only at biotic dilution 10^-1^ for both positive and negative cohesion on day 15 compared with the donors. Intermediate biotic dilution (i.e., AxB^-3^) presented cohesion values intermediate between the donors, whereas AxB^-5^ presented statistically similar cohesion values to the donors but lower negative cohesion on day 1 after MCC (Tukey’s HSD test, *P* < 0.05). In empirical AxC networks, positive cohesion was statistically higher than that of the donors at biotic dilution levels 10^-1^ and 10^-3^ on days 1 and 15, and was statistically similar or intermediate in the remaining treatments and time points. Negative cohesion for AxC treatments showed temporal fluctuations, but statistically higher values compared with the donors on day 1 across all dilution levels (Tukey’s HSD test, *P* < 0.05). Lastly, BxC treatments presented statistically similar positive cohesion compared with the donors, but lower values on day 1 in treatment BxC^-3^ and on day 5 in treatments BxC^-3^ and BxC^-5^. Negative cohesion was statistically similar or intermediate compared with the donors, except for treatment BxC^-3^ on days 1, 15, and 30 and BxC^-5^ on days 5 and 15, which presented lower values compared with the donors, whereas BxC^-5^ on day 1 showed higher negative cohesion (Tukey’s HSD test, *P* < 0.05).

In brief, BANM and BDS networks showed statistically intermediate or higher RN after MCC compared with the donors, except for BDS simulations in AxC^-1^ and BANM simulations in AxB^-3^ and AxC^-5^ (Tukey’s HSD test, *P* < 0.05). Empirical networks AxB showed statistically similar RN to the donors on days 1 and 5 for treatments AxB^-1^ and AxB^-3^ but lower values on days 15 and 30 compared with the donors and simulations (except for the AxB^-3^ BANM network) (Tukey’s HSD test, *P* < 0.05). Similarly, treatment BxC^-3^ showed higher RN on day 1, values similar to the donors on days 5 and 15, and lower RN on day 30, indicating a decrease in hierarchical structure over time after MCC (Tukey’s HSD test, *P* < 0.05). However, treatment AxC^-1^ showed temporal fluctuations in RN when compared with the donors, although it presented statistically lower RN compared with donor and simulation networks on days 1 and 15 after MCC (Tukey’s HSD test, *P* < 0.05). Empirical outcome networks AxC^-3^ and BxC^-1^ showed statistically lower RN compared with BANM and BDS simulations on days 1, 5, and 15 but values similar to or intermediate between both donors on day 30 (Tukey’s HSD test, *P* < 0.05). Interestingly, higher biotic dilution treatments (i.e., AxB^-5^, AxC^-5^, BxC^-5^) tended to present RN values intermediate between the donors, suggesting a more similar node architecture in empirical outcome networks compared with donor networks.

Coalescence effects on network stability across biotic dilution levels were evaluated using network robustness (weighted and unweighted), fragmentation, and vulnerability based on random node removal. Both BANM and BDS simulation networks presented robustness thresholds (i.e., robustness regression breakpoints) and fragmentation slopes (i.e., rates of network fragmentation) similar to or intermediate between the donors, except for AxB^-3^ and AxC^-1^, where the stochastic simulation presented lower fragmentation rates (95% confidence intervals, CI) (**Figures S3 and S4**). This indicates that simulated graphs predict the preservation of network stability metrics in MCC outcome networks. Likewise, empirical outcome networks showed robustness similar to one of the donors, but higher robustness for AxC^-5^ on day 15 and for BxC^-1^ on day 1 after MCC (95% CI) (**Figures S3 and S4**). However, network fragmentation showed different patterns across mixtures, biotic dilutions, and time. For treatment AxB, empirical biotic dilution 10^-1^ showed lower fragmentation slopes compared with the donors and both simulated networks (95% CI). Outcome AxB^-3^ showed fragmentation slopes similar to or intermediate between the donors on days 1 and 15 but higher values on days 5 and 30 after MCC, whereas AxB^-5^ showed a progressive increase in fragmentation slope from day 5 to day 30 (95% CI). In mixtures involving donor C, fragmentation showed similar rates compared with the donors for empirical mixtures AxC^-5^ and BxC^-3,-5^ across all time points. However, empirical treatments AxC^-1^ and AxC^-3^ showed decreased network fragmentation on days 1 and 15, whereas empirical BxC^-1^ showed a progressive increase in fragmentation, with lower rates on day 1, similar rates on days 5 and 15, and higher rates on day 30. For network vulnerability (i.e., maximum node vulnerability), empirical treatments showed increased vulnerability scores with increasing biotic dilution levels (**Table S5**). That is, low biotic dilution treatments (10^-1^) tended to show lower vulnerability compared with the donors and BANM simulation networks, particularly in treatment AxB^-1^, with scores below the computed range for BDS simulation networks.

*Network correlation threshold sensitivity*

Network properties were generally robust to variation in the correlation threshold used for network construction (**Figure S5**). Trajectories obtained using |*ρ*| ≥ 0.6 were highly consistent with those obtained using the original |*ρ*| ≥ 0.7 threshold, with statistically significant Spearman correlations ≥ 0.89 for all properties in AxB, ≥ 0.85 in AxC, and ≥ 0.73 in BxC (*P* < 0.05). Similarly, increasing the correlation stringency to |*ρ*| ≥ 0.8 preserved the trajectories of most network properties. Structural properties, including the number of edges and nodes, degree, clustering, fragmentation, cohesion, and relative modularity, remained strongly correlated with those obtained at |*ρ*| ≥ 0.7 across all three coalescences (*ρ* ≥ 0.71; *P* < 0.05).

Network robustness was comparatively more sensitive to threshold selection. In AxB, weighted and unweighted robustness remained strongly correlated across thresholds (*ρ* = 0.86 – 0.94; *P* < 0.05). In AxC, correlations remained significant but were weaker for weighted robustness at |*ρ*| ≥ 0.8 (*ρ* = 0.64, *P* < 0.05). The greatest sensitivity occurred in BxC, where weighted and unweighted robustness at |*ρ*| ≥ 0.8 were not significantly correlated with the corresponding trajectories at |*ρ*| ≥ 0.7 (*ρ* = 0.23 and 0.15, respectively; *P* > 0.05). Unweighted robustness in BxC also showed lower consistency between |*ρ*| ≥ 0.6 and |*ρ*| ≥ 0.7 (*ρ* = 0.56, *P* > 0.05).

**Supplementary Tables**

**Table S1.** Glossary of co-occurrence network topological metrics used in the manuscript.

| **Metric** | **Definition** |
| --- | --- |
| node | is an entity in the network (e.g., a microbial strain, OTU). |
| edge | is a significant correlation between nodes. It can be positive or negative, weighed or unweighted. |
| degree | of a node is its number of edges. |
| clustering coefficient | of node is the degree to which the node is connected to their neighbors. |
| density | of network is the proportion of realized links in all possible ones. |
| size | of a network is its total number of nodes. |
| connectivity | of a network is its total number of edges. |
| average degree | of network is the average nodes’ degree. |
| average cohesion | of network is the abundance-weighted positive or negative correlations, corrected by null model. |
| positive: negative ratio | of network is the ratio of the number of positive and negative edges. |
| average path length | of network is the average shortest path between all the pairs of nodes. |
| relative modularity (RM) | of network measures its degree of compartmentation into different modules corrected by random expectation. |
| relative nestedness (RN) | of network measures the degree of hierarchical organization or the tendency of nodes with few edges correlate with nodes with nodes with many correlations corrected by random expectation. |
| vulnerability | of network measures the maximum impact of a node removal on network efficiency. |
| robustness | of network measures the nodes persistence after random removal, abundance weighted or unweighted. |
| fragmentation | of network measures the number of disconnected compounds after random nodes removal. |

**Table S2.** Topological parameters estimated for each co-occurrence network across donor and outcome communities. Different lowercase letters indicate statistically significant differences among outcome networks within the same mixture and biotic dilution level (Tukey’s HSD, P < 0.05). Treatments sharing at least one letter are not significantly different. Donor networks were compared with their respective mixture outcomes, and the letters refer to comparisons performed **within the treatment indicated in brackets** (e.g., comparisons among treatments within AxB^-1^ or within AxC^-1^). For the BDS simulations, values are presented as the mean (μ) of 20 simulated networks, and the values in brackets represent the observed range (minimum–maximum) across those simulations.

| Treatment | Density | Clustering Coefficient | Degree | Ratio Positive: Negative | RM | RN | Positive cohesion | Negative cohesion |
| --- | --- | --- | --- | --- | --- | --- | --- | --- |
| Donor A^-1^ | 0.0122 | 0.17  cd [AxB^-1^];  b [AxC^-1^] | 2.851  c [AxB^-1^];  c [AxC^-1^] | 1.72 | 0.18  f [AxB^-1^];  c [AxC^-1^] | -0.294  cd [AxB^-1^];  c [AxC^-1^] | 0.447  d [AxB^-1^];  cd [AxC^-1^] | -0.403  a [AxB^-1^];  ab [AxC^-1^] |
| Donor A^-3^ | 0.0102 | 0.132  b [AxB^-3^];  c [AxC^-3^] | 1.948  c [AxB^-3^];  e [AxC^-3^] | 2.28 | 0.06  f [AxB^-3^];  g [AxC^-3^] | -0.065  a [AxB^-3^];  a [AxC^-3^] | 0.404  d [AxB^-3^];  de [AxC^-3^] | -0.411  ab [AxB^-3^];  b [AxC^-3^] |
| Donor A^-5^ | 0.0167 | 0.09  c [AxB^-5^];  d [AxC^-5^] | 1.733  b [AxB^-5^];  c [AxC^-5^] | 1.22 | 0.04  e [AxB^-5^];  f[AxC^-5^] | -0.101  a [AxB^-5^];  a [AxC^-5^] | 0.418  d [AxB^-5^];  c [AxC^-5^] | -0.424  bc [AxB^-5^];  c [AxC^-5^] |
| Donor B^-1^ | 0.0157 | 0.098  d [AxB^-1^];  d [BxC^-1^] | 1.884  c [AxB^-1^];  e [BxC^-1^] | 1.65 | 0.06  g [AxB^-1^];  g [BxC^-1^] | -0.194  a [AxB^-1^];  b [BxC^-1^] | 0.490  cd [AxB^-1^];  b [BxC^-1^] | -0.505  b [AxB^-1^];  b [BxC^-1^] |
| Donor B^-3^ | 0.0189 | 0.152  b [AxB^-3^];  b [BxC^-3^] | 2.172  bc [AxB^-3^];  d [BxC^-3^] | 1.52 | 0.02  g [AxB^-3^];  g [BxC^-3^] | -0.259  d [AxB^-3^];  de [BxC^-3^] | 0.490  a [AxB^-3^];  a [BxC^-3^] | -0.506  e [AxB^-3^];  e [BxC^-3^] |
| Donor B^-5^ | 0.0285 | 0.187  abc [AxB^-5^];  b [BxC^-5^] | 3.083  ab [AxB^-5^];  c [BxC^-5^] | 1.95 | 0.11  d [AxB^-5^];  e [BxC^-5^] | -0.481  e [AxB^-5^];  f [BxC^-5^] | 0.534  b [AxB^-5^];  ab [BxC^-5^] | -0.535  f [AxB^-5^];  f [BxC^-5^] |
| Donor C^-1^ | 0.0127 | 0.151  b [AxC^-1^];  cd [BxC^-1^] | 2.877  c [AxC^-1^];  de [BxC^-1^] | 1.26 | 0.14  d [AxC^-1^];  f [BxC^-1^] | -0.290  c [AxC^-1^];  d [BxC^-1^] | 0.421  de [AxC^-1^];  de [BxC^-1^] | -0.444  c [AxC^-1^];  a [BxC^-1^] |
| Donor C^-3^ | 0.0125 | 0.239  ab [AxC^-3^];  ab [BxC^-3^] | 3.479  cd [AxC^-3^];  ab [BxC^-3^] | 1.31 | 0.20  d [AxC^-3^];  de [BxC^-3^] | -0.247  d [AxC^-3^];  cd [BxC^-3^] | 0.448  c [AxC^-3^];  bc [BxC^-3^] | -0.420  b [AxC^-3^];  d [BxC^-3^] |
| Donor C^-5^ | 0.0182 | 0.282  ab [AxC^-5^];  ab [BxC^-5^] | 4.190  ab [AxC^-5^];  c [BxC^-5^] | 1.64 | 0.36  b [AxC^-5^];  c [BxC^-5^] | -0.263  c [AxC^-5^];  b [BxC^-5^] | 0.520  a [AxC^-5^];  ab [BxC^-5^] | -0.409  bc [AxC^-5^];  cd [BxC^-5^] |
| AxB^-1^ |  |  |  |  |  |  |  |  |
| BANM simulation | 0.0419 | 0.191 bcd | 3.476 c | 8.13 | 0.26 e | -0.224 ab | 0.562 ab | -0.565 c |
| BDS simulation | μ=0.012  [0.008‚0.016] | μ=0.215 bc  [0.215‚0.215] | μ=4.941 c  [2.878‚7.591] | μ=2.02  [1.51‚2.52] | μ=0.416 d  [0.123,0.706] | μ=-0.304 d  [-0.515,0.012] | μ=0.470 cd  [0.388,0.519] | μ=-0.466 b  [-0.512,-0.383] |
| day 1 | 0.0154 | 0.245 b | 5.181 c | 1.97 | 0.46 c | -0.262 bc | 0.469 cd | -0.465 b |
| day 5 | 0.0411 | 0.382 a | 21.910 a | 1.32 | 1.08 b | -0.230 ab | 0.485 cd | -0.492 b |
| day 15 | 0.0299 | 0.356 a | 17.091 b | 2.35 | 1.33 a | -0.409 e | 0.612 a | -0.605 c |
| day 30 | 0.0304 | 0.339 a | 16.514 b | 1.39 | 1.38 a | -0.441 e | 0.508 bc | -0.477 b |
| AxB^-3^ |  |  |  |  |  |  |  |  |
| BANM simulation | 0.0667 | 0.14 b | 1.600 c | 2.33 | 0.20 c | -0.690 f | 0.430 c | -0.480 d |
| BDS simulation | μ=0.009  [0.007‚0.012] | μ=0.161 b  [0.161‚0.161] | μ=2.656 b  [2.242‚3.361] | μ=1.73  [1.21‚2.18] | μ=0.157 d [0.049,0.291] | μ=-0.195 b  [-0.427,0.034] | μ=0.408 d  [0.386,0.435] | μ=-0.407 a  [-0.441,-0.387] |
| day 1 | 0.0103 | 0.128 b | 2.030 c | 1.36 | 0.05 f | -0.210 bc | 0.437 bc | -0.425 b |
| day 5 | 0.0172 | 0.14 b | 2.423 bc | 1.39 | 0.08 e | -0.222 c | 0.446 bc | -0.453 c |
| day 15 | 0.0138 | 0.228 a | 3.944 a | 1.26 | 0.34 b | -0.403 e | 0.475 a | -0.474 cd |
| day 30 | 0.0142 | 0.204 ab | 4.069 a | 2.02 | 0.37 a | -0.401 e | 0.452 b | -0.463 cd |
| AxB^-5^ |  |  |  |  |  |  |  |  |
| BANM simulation | 0.0567 | 0.075 c | 1.586 b | 0.77 | -0.05 f | -0.160 b | 0.613 a | -0.603 g |
| BDS simulation | μ=0.015  [0.009‚0.023] | μ=0.168 bc  [0.168‚0.168] | μ=3.381 a  [2.273‚4.450] | μ=1.70  [1.05‚2.67] | μ=0.152 c  [0.002,0.353] | μ=-0.334 d  [-0.523,-0.069] | μ=0.465 c  [0.430,0.511] | μ=-0.451 d  [-0.489,-0.420] |
| day 1 | 0.0137 | 0.125 bc | 1.656 b | 1.59 | 0.04 e | -0.292 c | 0.398 d | -0.395 a |
| day 5 | 0.0115 | 0.176 abc | 2.350 b | 1.57 | 0.12 d | -0.144 b | 0.426 d | -0.411 ab |
| day 15 | 0.0147 | 0.238 a | 3.831 a | 1.44 | 0.28 b | -0.350 d | 0.469 c | -0.449 cd |
| day 30 | 0.0169 | 0.209 ab | 3.444 a | 1.58 | 0.35 a | -0.129 ab | 0.511 b | -0.500 e |
| AxC^-1^ |  |  |  |  |  |  |  |  |
| BANM simulation | 0.0423 | 0.239 ab | 2.414 c | 13.00 | 0.14 d | -0.199 b | 0.454 c | -0.514 e |
| BDS simulation | μ=0.013  [0.010‚0.021] | μ=0.246 a  [0.246‚0.246] | μ=5.009 b  [3.716‚8.502] | μ=1.80  [1.21‚2.38] | μ=0.427 b  [0.241,0.776] | μ=-0.329 d  [-0.458,-0.076] | μ=0.414 e  [0.381,0.461] | μ=-0.410 b  [-0.457,-0.383] |
| day 1 | 0.0209 | 0.283 a | 6.482 a | 1.08 | 0.44 b | -0.365 e | 0.621 a | -0.688 f |
| day 5 | 0.0099 | 0.187 b | 2.567 c | 1.86 | 0.13 d | -0.164 a | 0.401 e | -0.392 a |
| day 15 | 0.0192 | 0.284 a | 7 a | 1.35 | 0.57 a | -0.372 e | 0.492 b | -0.469 d |
| day 30 | 0.0092 | 0.188 b | 3.379 c | 1.25 | 0.17 c | -0.291c | 0.428 cde | -0.399 ab |
| AxC^-3^ |  |  |  |  |  |  |  |  |
| BANM simulation | 0.0235 | 0.16 bc | 2.303 de | 2.35 | 0.15 e | -0.177 b | 0.447 c | -0.418 b |
| BDS simulation | μ=0.009  [0.008‚0.010] | μ=0.212 b  [0.212‚0.212] | μ=3.464 cd  [2.930‚4.025] | μ=1.23  [1.11‚1.42] | μ=0.218 c [0.100,0.324] | μ=-0.221 c  [-0.366,-0.074] | μ=0.416 d  [0.395,0.447] | μ=-0.386 a  [-0.406,-0.369] |
| day 1 | 0.0246 | 0.3 a | 8.768 a | 1.25 | 0.59 a | -0.439 f | 0.497 a | -0.510 d |
| day 5 | 0.0110 | 0.229 b | 3.547 c | 1.28 | 0.22 c | -0.262 d | 0.465 bc | -0.414 b |
| day 15 | 0.0189 | 0.254 ab | 6.201 b | 1.19 | 0.42 b | -0.396 e | 0.478 ab | -0.471 c |
| day 30 | 0.0073 | 0.139 c | 2.287 e | 1.60 | 0.08 f | -0.164 b | 0.396 e | -0.393 a |
| AxC^-5^ |  |  |  |  |  |  |  |  |
| BANM simulation | 0.0452 | 0.169 cd | 3.978 ab | 12.62 | 0.28 d | -0.539 f | 0.530 a | -0.361 a |
| BDS simulation | μ=0.014  [0.012‚0.016] | μ=0.252 bc  [0.252‚0.252] | μ=4.460 a  [4.096‚4.890] | μ=1.46  [1.20‚1.80] | μ=0.393 a  [0.282,0.526] | μ=-0.319 d  [-0.444,-0.099] | μ=0.448 b  [0.420,0.475] | μ=-0.399 b  [-0.424,-0.372] |
| day 1 | 0.0754 | 0.39 a | 4.373 ab | 42.00 | 0.23 e | -0.125 a | 0.528 a | -0.541 e |
| day 5 | 0.0127 | 0.227 bc | 4.717 a | 1.82 | 0.37 b | -0.413 e | 0.421 c | -0.361 a |
| day 15 | 0.0161 | 0.227 bc | 3.359 b | 1.19 | 0.28 d | -0.171 b | 0.518 a | -0.397 b |
| day 30 | 0.0210 | 0.256 abc | 3.485 b | 1.47 | 0.32 c | -0.189 b | 0.537 a | -0.514 d |
| BxC^-1^ |  |  |  |  |  |  |  |  |
| BANM simulation | 0.0406 | 0.219 bc | 4.059 cd | 1.70 | 0.22 e | -0.073 a | 0.571 a | -0.610 c |
| BDS simulation | μ=0.012  [0.008‚0.020] | μ=0.229 b  [0.229‚0.229] | μ=4.587 c  [3.093‚7.090] | μ=1.46  [1.04‚1.95] | μ=0.324 d [0.144,0.653] | μ=-0.249 c  [-0.425,-0.039] | μ=0.458 c  [0.414,0.518] | μ=-0.455 a  [-0.511,-0.413] |
| day 1 | 0.0223 | 0.291a | 8.082 a | 1.85 | 0.80 a | -0.345 e | 0.452 cd | -0.448 a |
| day 5 | 0.0204 | 0.299 a | 6.821 b | 1.45 | 0.59 b | -0.354 e | 0.506 b | -0.509 b |
| day 15 | 0.0144 | 0.228b | 4.521 c | 1.54 | 0.41 c | -0.353 e | 0.442 cde | -0.454 a |
| day 30 | 0.0104 | 0.177 bcd | 2.933 de | 1.73 | 0.17 f | -0.221 b | 0.410 e | -0.436 a |
| BxC^-3^ |  |  |  |  |  |  |  |  |
| BANM simulation | 0.0204 | 0.151 b | 2.283 cd | 2.00 | 0.16 f | -0.040 a | 0.454 b | -0.403 bcd |
| BDS simulation | μ=0.010  [0.009‚0.011] | μ=0.234 ab  [0.234‚0.234] | μ=3.791 a  [3.469‚4.257] | μ=1.19  [1.03‚1.53] | μ=0.258 b [0.160,0.351] | μ=-0.243 c  [-0.375,-0.097] | μ=0.419 d  [0.387,0.463] | μ=-0.387 b  [-0.412,-0.366] |
| day 1 | 0.0107 | 0.218 ab | 3.014 bcd | 1.68 | 0.18 e | -0.220 b | 0.426 cd | -0.362 a |
| day 5 | 0.0117 | 0.263 a | 3.841 a | 1.81 | 0.35 a | -0.277 e | 0.409 d | -0.411 cd |
| day 15 | 0.0094 | 0.214 ab | 3.217 abc | 1.39 | 0.24 c | -0.303 e | 0.441 bc | -0.385 b |
| day 30 | 0.0093 | 0.222 ab | 3.366 ab | 1.46 | 0.22 d | -0.278 f | 0.428 cd | -0.394 bc |
| BxC^-5^ |  |  |  |  |  |  |  |  |
| BANM simulation | 0.0304 | 0.208 ab | 3.521 c | 4.02 | 0.46 b | -0.072 a | 0.536 ab | -0.422 d |
| BDS simulation | μ=0.016  [0.012‚0.022] | μ=0.271 ab  [0.271‚0.271] | μ=5.435 b  [4.258‚7.418] | μ=1.43  [1.24‚1.70] | μ=0.542 a  [0.368,0.807] | μ=-0.331 d  [-0.460,-0.192] | μ=0.446 d  [0.409,0.502] | μ=-0.393 bc  [-0.433,-0.362] |
| day 1 | 0.0240 | 0.272 ab | 6.215 ab | 1.23 | 0.45 b | -0.374 e | 0.555 a | -0.499 e |
| day 5 | 0.0125 | 0.224 ab | 4.212 c | 1.38 | 0.33 d | -0.307 c | 0.441 d | -0.375 ab |
| day 15 | 0.0181 | 0.292 a | 6.124 ab | 1.13 | 0.50 b | -0.372 e | 0.477 c | -0.370 a |
| day 30 | 0.0170 | 0.273 ab | 6.442 a | 1.39 | 0.56 a | -0.338 d | 0.511 bc | -0.396 bcd |

**Table S3.** Chi-square tests comparing the proportions of persistent nodes among BANM simulations, BDS simulations, and empirical networks following MCC. For the BDS simulations, values are presented as the mean (μ) of 20 simulated networks, and the values in brackets represent the observed range (minimum–maximum) across those simulations.

| Treatment | Nodes shared with A | Nodes shared with B | Nodes shared with A and B | Emergent nodes | Donors derived (%) | BANM χ² test | Monte Carlo p-value | BDS χ² test | Monte Carlo p-value |
| --- | --- | --- | --- | --- | --- | --- | --- | --- | --- |
| AxB^-1^ |  |  |  |  |  |  |  |  |  |
| BANM simulation | 20.24% | 30.95% | 3.57% | 45.24% | 54.76% |  |  |  |  |
| BDS simulation | μ=24.1%  [18.6, 34] | μ=14.2 %  [12.2, 19.8] | μ=3.3%  [2.3, 5.2] | μ=58.4%  [42.2, 66.0] | μ=45.9%  [34, 57.8] | 20.29 | 0.00090 |  |  |
| day 1 | 16.32% | 15.73% | 3.56% | 64.39% | 35.61% | 12.98 | 0.00482 | 9.43 | 0.02350 |
| day 5 | 15.73% | 11.42% | 3.18% | 69.66% | 30.34% | 27.13 | 0.00005 | 24.35 | 0.00020 |
| day 15 | 14.51% | 10.66% | 1.92% | 72.90% | 27.10% | 33.32 | 0.00003 | 42.27 | 0.00010 |
| day 30 | 12.84% | 8.26% | 2.75% | 76.15% | 23.85% | 45.91 | 0.00001 | 61.83 | 0.00010 |
| AxB^-3^ |  |  |  |  |  |  |  |  |  |
| BANM simulation | 12.00% | 56.00% | 8.00% | 24.00% | 76.00% |  |  |  |  |
| BDS simulation | μ=29.0%  [24.0, 33.6] | μ=21.0%  [17.4, 24.1] | μ=4.1%  [3.1, 5.5] | μ=45.9  [38.4, 53.7] | μ=53.95%  [46.3, 61.6] | 20.63 | 0.00070 |  |  |
| day 1 | 21.21% | 16.16% | 3.03% | 59.60% | 40.40% | 24.60 | 0.00016 | 13.99 | 0.00330 |
| day 5 | 23.24% | 16.90% | 2.82% | 57.04% | 42.96% | 21.57 | 0.00035 | 6.71 | 0.07779 |
| day 15 | 16.72% | 15.68% | 3.48% | 64.11% | 35.89% | 27.38 | 0.00009 | 36.92 | 0.00010 |
| day 30 | 13.89% | 13.89% | 2.43% | 69.79% | 30.21% | 33.48 | 0.00004 | 62.78 | 0.00010 |
| AxB^-5^ |  |  |  |  |  |  |  |  |  |
| BANM simulation | 17.24% | 72.41% | 3.45% | 6.90% | 93.10% |  |  |  |  |
| BDS simulation | μ=17.4%  [14.7, 20.9] | μ=24.0%  [18.9, 31.3] | μ=3.0%  [2.3, 4.2] | μ=55.6%  [46.2, 62.2] | μ=45.8%  [37.8, 53.8] | 41.04 | 0. 00001 |  |  |
| day 1 | 9.84% | 18.85% | 1.64% | 69.67% | 30.33% | 40.62 | 0.00001 | 9.69 | 0.02260 |
| day 5 | 11.65% | 15.53% | 2.43% | 70.39% | 29.61% | 53.60 | 0.00001 | 16.66 | 0.00110 |
| day 15 | 11.11% | 15.33% | 1.92% | 71.65% | 28.35% | 58.36 | 0.00001 | 24.51 | 0.00030 |
| day 30 | 9.27% | 14.15% | 2.44% | 74.15% | 25.85% | 59.50 | 0.00001 | 26.64 | 0.00010 |
|  | Nodes shared with A | Nodes shared with C | Nodes shared with A and C | Emergent nodes | Donors derived (%) |  |  |  |  |
| AxC^-1^ |  |  |  |  |  |  |  |  |  |
| BANM simulation | 13.79% | 58.62% | 15.52% | 12.07% | 87.93% |  |  |  |  |
| BDS simulation | μ=21.7%  [19.8, 24.6] | μ=30.7%  [26.8, 33.7] | μ=6.8%  [5.7, 8.5] | μ=40.8%  [35.1, 46.3] | μ=59.3%  [53.7, 64.9] | 34.27 | 0. 00001 |  |  |
| day 1 | 5.79% | 37.62% | 8.68% | 47.91% | 52.09% | 26.91 | 0.00005 | 45.49 | 0.00010 |
| day 5 | 8.05% | 32.95% | 8.81% | 50.19% | 49.81% | 28.11 | 0.00002 | 29.29 | 0.00010 |
| day 15 | 6.56% | 32.79% | 6.83% | 53.83% | 46.17% | 35.53 | 0.00003 | 52.59 | 0.00010 |
| day 30 | 7.63% | 31.34% | 7.36% | 53.68% | 46.32% | 34.88 | 0.00001 | 46.87 | 0.00010 |
| AxC^-3^ |  |  |  |  |  |  |  |  |  |
| BANM simulation | 15.15% | 66.67% | 11.11% | 7.07% | 92.93% |  |  |  |  |
| BDS simulation | μ=19.4%  [16.1, 21.5] | μ=39.9%  [37.5, 42.3] | μ=8.0%  [7.0, 9.3] | μ=32.7%  [27.7, 37.0] | μ=67.65%  [63, 72.3] | 39.29 | 0. 00001 |  |  |
| day 1 | 10.36% | 37.25% | 7.56% | 44.82% | 55.18% | 48.25 | 0.00001 | 30.35 | 0.00010 |
| day 5 | 5.59% | 38.20% | 8.39% | 47.83% | 52.17% | 56.01 | 0.00001 | 52.68 | 0.00010 |
| day 15 | 7.90% | 36.47% | 7.29% | 48.33% | 51.67% | 54.89 | 0.00001 | 46.21 | 0.00010 |
| day 30 | 11.15% | 35.99% | 7.01% | 45.86% | 54.14% | 49.91 | 0.00001 | 28.00 | 0.00010 |
| AxC^-5^ |  |  |  |  |  |  |  |  |  |
| BANM simulation | 8.99% | 73.03% | 8.99% | 8.99% | 91.01% |  |  |  |  |
| BDS simulation | μ=10.9%  [9.6, 13.9] | μ=43.3%  [39.1, 52.6] | μ=4.5%  [4.0, 5.3] | μ=41.3%  [28.8, 46.1] | μ=62.55%  [53.9, 71.2] | 44.80 | 0. 00001 |  |  |
| day 1 | 3.39% | 49.15% | 15.25% | 32.20% | 67.80% | 16.53 | 0.00061 | 19.30 | 0.00090 |
| day 5 | 5.66% | 34.77% | 3.77% | 55.80% | 44.20% | 63.91 | 0.00001 | 32.06 | 0.00010 |
| day 15 | 7.18% | 35.89% | 6.22% | 50.72% | 49.28% | 47.69 | 0.00001 | 10.39 | 0.01670 |
| day 30 | 10.78% | 32.34% | 4.19% | 52.69% | 47.31% | 52.73 | 0.00001 | 9.31 | 0.02380 |
|  | Nodes shared with B | Nodes shared with C | Nodes shared with B and C | Emergent nodes | Donors derived (%) |  |  |  |  |
| BxC^-1^ |  |  |  |  |  |  |  |  |  |
| BANM simulation | 9.90% | 69.31% | 3.96% | 16.83% | 83.17% |  |  |  |  |
| BDS simulation | μ=14.3%  [12.1, 16.4] | μ=37.4%  [32.5, 43.0] | μ=2.6%  [2.1, 3.1] | μ=45.7%  [39.9, 50] | μ= 55.05%  [50, 60.1] | 47.53 | 0.00001 |  |  |
| day 1 | 9.34% | 35.99% | 2.75% | 51.92% | 48.08% | 42.69 | 0.00001 | 9.04 | 0.02850 |
| day 5 | 8.33% | 35.42% | 2.98% | 53.27% | 46.73% | 44.07 | 0.00001 | 12.49 | 0.00750 |
| day 15 | 6.67% | 38.73% | 1.90% | 52.70% | 47.30% | 40.27 | 0.00001 | 16.33 | 0.00110 |
| day 30 | 5.30% | 37.81% | 2.83% | 54.06% | 45.94% | 42.05 | 0.00001 | 19.86 | 0.00060 |
| BxC^-3^ |  |  |  |  |  |  |  |  |  |
| BANM simulation | 12.39% | 58.41% | 12.39% | 16.81% | 83.19% |  |  |  |  |
| BDS simulation | μ=14.7%  [12.1, 17.8] | μ=44.7%  [40.7, 48.8] | μ=5.0%  [3.9, 5.9] | μ=35.6%  [28.7, 41.6] | μ=64.85%  [58.4, 71.3] | 28.36 |  |  |  |
| day 1 | 8.10% | 44.37% | 4.58% | 42.96% | 57.04% | 27.70 | 0.00001 | 12.26 | 0.00600 |
| day 5 | 7.62% | 40.24% | 5.18% | 46.95% | 53.05% | 34.00 | 0.00001 | 23.20 | 0.00010 |
| day 15 | 6.09% | 40.00% | 4.64% | 49.28% | 50.72% | 40.44 | 0.00001 | 35.37 | 0.00010 |
| day 30 | 6.34% | 38.57% | 4.96% | 50.14% | 49.86% | 41.64 | 0.00001 | 39.50 | 0.00010 |
| BxC^-5^ |  |  |  |  |  |  |  |  |  |
| BANM simulation | 16.24% | 59.83% | 5.98% | 17.95% | 82.05% |  |  |  |  |
| BDS simulation | μ=15.8%  [12.5, 18.4] | μ=42.4%  [39.7, 45.9] | μ=2.6%  [1.8, 3.0] | μ=39.2%  [33.2, 43.4] | μ=61.7%  [56.6, 66.8] | 26.80 | 0.00001 |  |  |
| day 1 | 10.00% | 43.08% | 3.08% | 43.85% | 56.15% | 24.15 | 0.00005 | 7.02 | 0.07099 |
| day 5 | 11.21% | 38.05% | 2.06% | 48.67% | 51.33% | 35.69 | 0.00001 | 13.31 | 0.00620 |
| day 15 | 7.96% | 37.17% | 2.36% | 52.51% | 47.49% | 43.57 | 0.00001 | 29.19 | 0.00010 |
| day 30 | 9.74% | 31.32% | 2.37% | 56.58% | 43.42% | 54.23 | 0.00001 | 46.01 | 0.00010 |

**Table S4.** Chi-square tests comparing the proportions of persistent edges among BANM simulations, BDS simulations, and empirical networks following MCC. For the BDS simulations, values are presented as the mean (μ) of 20 simulated networks, and the values in brackets represent the observed range (minimum–maximum) across those simulations.

| Treatment | Edges shared with A | Edges shared with B | Edges shared with A and B | New edges | Donors derived (%) | BANM χ² test | Fisher’s exact p-value | BDS χ² test | Fisher’s exact p-value |
| --- | --- | --- | --- | --- | --- | --- | --- | --- | --- |
| AxB^-1^ |  |  |  |  |  |  |  |  |  |
| BANM simulation | 2.74% | 2.05% | 0.00% | 95.21% | 4.79% |  |  |  |  |
| BDS simulation | μ=3.9%  [1.2, 11.5] | μ=1.1%  [0.3, 2.6] | μ=0%  [0,0] | μ=94.9%  [86.7, 98.5] | μ=7.4%  [1.5, 13.3] | 2.10 | 0.302119 |  |  |
| day 1 | 0.11% | 0.23% | 0.00% | 99.66% | 0.34% | 26.32 | 0.000187 | 29.06 | >0.00001 |
| day 5 | 0.02% | 0.36% | 0.00% | 99.62% | 0.38% | 137.14 | >0.00001 | 192.51 | >0.00001 |
| day 15 | 0.04% | 0.27% | 0.00% | 99.69% | 0.31% | 101.19 | >0.00001 | 164.87 | >0.00001 |
| day 30 | 0.07% | 0.00% | 0.00% | 99.93% | 0.07% | 159.85 | >0.00001 | 171.78 | >0.00001 |
| AxB^-3^ |  |  |  |  |  |  |  |  |  |
| BANM simulation | 5.00% | 10.00% | 0.00% | 85.00% | 15.00% |  |  |  |  |
| BDS simulation | μ=4%  [1.7, 6.5] | μ=5.8%  [2.6, 10.7] | μ=0%  [0,0] | μ=90.2%  [84.1, 94.7] | μ= 10.6%  [5.3, 15.9] | 0.79 | 0.345440 |  |  |
| day 1 | 0.50% | 0.50% | 0.00% | 99.00% | 1.00% | 16.54 | 0.005695 | 16.97 | 0.00001 |
| day 5 | 0.00% | 0.00% | 0.00% | 100.00% | 0.00% | 26.21 | 0.000982 | 18.14 | >0.00001 |
| day 15 | 0.18% | 0.00% | 0.00% | 99.82% | 0.18% | 70.20 | 0.000134 | 57.16 | >0.00001 |
| day 30 | 0.34% | 0.00% | 0.00% | 99.66% | 0.34% | 67.50 | 0.000121 | 57.12 | >0.00001 |
| AxB^-5^ |  |  |  |  |  |  |  |  |  |
| BANM simulation | 0.00% | 13.04% | 0.00% | 86.96% | 13.04% |  |  |  |  |
| BDS simulation | μ=1.2%  [0.6, 2.4] | μ=8.4%  [2, 15] | μ=0%  [0,0] | μ=90.4%  [84, 96.7] | μ=9.65%  [3.3, 16] | 0.83 | 0.579605 |  |  |
| day 1 | 0.00% | 0.00% | 0.00% | 100.00% | 0.00% | 8.54 | 0.005711 | 10.94 | 0.00055 |
| day 5 | 0.00% | 0.41% | 0.00% | 99.59% | 0.41% | 14.84 | 0.002178 | 23.98 | >0.00001 |
| day 15 | 0.00% | 0.60% | 0.00% | 99.40% | 0.60% | 20.05 | 0.001369 | 47.33 | >0.00001 |
| day 30 | 0.00% | 0.00% | 0.00% | 100.00% | 0.00% | 31.40 | 0.000202 | 38.11 | >0.00001 |
| Treatment | Edges shared with A | Edges shared with C | Edges shared with A and C | New edges | Donors derived (%) |  |  |  |  |
| AxC^-1^ |  |  |  |  |  |  |  |  |  |
| BANM simulation | 7.14% | 15.71% | 0.00% | 77.14% | 22.86% |  |  |  |  |
| BDS simulation | μ=2.8%  [2, 4.4] | μ=4.5%  [2.4, 5.7] | μ=0%  [0,0] | μ=92.6%  [90.4, 95] | μ=7.3%  [5, 9.6] | 26.00 | 0.00006 |  |  |
| day 1 | 0.10% | 1.49% | 0.00% | 98.41% | 1.59% | 116.32 | >0.00001 | 48.92 | >0.00001 |
| day 5 | 0.60% | 1.49% | 0.00% | 97.91% | 2.09% | 46.65 | >0.00001 | 13.25 | 0.00034 |
| day 15 | 0.00% | 1.25% | 0.00% | 98.75% | 1.25% | 164.17 | >0.00001 | 69.34 | >0.00001 |
| day 30 | 0.00% | 0.16% | 0.00% | 99.84% | 0.16% | 135.10 | >0.00001 | 46.17 | >0.00001 |
| AxC^-3^ |  |  |  |  |  |  |  |  |  |
| BANM simulation | 3.51% | 18.42% | 0.00% | 78.07% | 21.93% |  |  |  |  |
| BDS simulation | μ=2.6%  [1.6, 4] | μ=10.4%  [7.2, 15.9] | μ=0%  [0,0] | μ=86.9%  [81, 91] | μ= 14%  [9, 19] | 8.65 | 0.013435 |  |  |
| day 1 | 0.19% | 0.70% | 0.00% | 99.11% | 0.89% | 207.89 | >0.00001 | 195.96 | >0.00001 |
| day 5 | 0.00% | 0.35% | 0.00% | 99.65% | 0.35% | 117.06 | >0.00001 | 79.35 | >0.00001 |
| day 15 | 0.00% | 0.29% | 0.00% | 99.71% | 0.29% | 199.91 | >0.00001 | 142.32 | >0.00001 |
| day 30 | 0.56% | 0.28% | 0.00% | 99.16% | 0.84% | 71.27 | >0.00001 | 46.44 | >0.00001 |
| AxC^-5^ |  |  |  |  |  |  |  |  |  |
| BANM simulation | 1.13% | 33.90% | 0.00% | 64.97% | 35.03% |  |  |  |  |
| BDS simulation | μ= 0.3%  [0, 1] | μ=15.9%  [12.3, 23.1] | μ= 0  [0,0] | μ=83.8%  [75.8, 87.6] | μ=18.3%  [12.4, 24.3] | 48.54 | >0.00001 |  |  |
| day 1 | 0.00% | 1.55% | 0.00% | 98.45% | 1.55% | 50.57 | >0.00001 | 19.89 | >0.00001 |
| day 5 | 0.00% | 0.57% | 0.00% | 99.43% | 0.57% | 293.20 | >0.00001 | 152.35 | >0.00001 |
| day 15 | 0.00% | 0.85% | 0.00% | 99.15% | 0.85% | 127.31 | >0.00001 | 59.30 | >0.00001 |
| day 30 | 0.00% | 1.03% | 0.00% | 98.97% | 1.03% | 106.38 | >0.00001 | 48.05 | >0.00001 |
| Treatment | Edges shared with B | Edges shared with C | Edges shared with B and C | New edges | Donors derived (%) |  |  |  |  |
| BxC^-1^ |  |  |  |  |  |  |  |  |  |
| BANM simulation | 0.00% | 15.61% | 0.00% | 84.39% | 15.61% |  |  |  |  |
| BDS simulation | μ=0.8%  [0.3, 1.7] | μ=8.5%  [5.8, 11.4] | μ=0%  [0,0] | μ=90.7%  [87.7, 93.6] | μ=9.35%  [6.4, 12.3] | 15.01 | 0.001729 |  |  |
| day 1 | 0.20% | 0.07% | 0.00% | 99.73% | 0.27% | 225.47 | >0.00001 | 138.62 | >0.00001 |
| day 5 | 0.00% | 1.48% | 0.00% | 98.52% | 1.48% | 95.28 | >0.00001 | 79.84 | >0.00001 |
| day 15 | 0.00% | 0.56% | 0.00% | 99.44% | 0.56% | 91.62 | >0.00001 | 62.69 | >0.00001 |
| day 30 | 0.24% | 1.20% | 0.00% | 98.55% | 1.45% | 51.14 | >0.00001 | 29.49 | >0.00001 |
| BxC^-3^ |  |  |  |  |  |  |  |  |  |
| BANM simulation | 2.33% | 14.73% | 0.00% | 82.95% | 17.05% |  |  |  |  |
| BDS simulation | μ=2.95  [1, 5.2] | μ=14.2%  [10.3, 18.1] | μ=0%  [0,0] | μ=82.9%  [77.9, 86.9] | μ=17.6%  [13.1, 22.1] | 0.18 | 0.958286 |  |  |
| day 1 | 0.00% | 0.70% | 0.00% | 99.30% | 0.70% | 62.11 | >0.00001 | 80.27 | >0.00001 |
| day 5 | 0.48% | 0.63% | 0.00% | 98.89% | 1.11% | 77.65 | >0.00001 | 112.05 | >0.00001 |
| day 15 | 0.00% | 0.54% | 0.00% | 99.46% | 0.54% | 81.38 | >0.00001 | 105.95 | >0.00001 |
| day 30 | 0.00% | 0.00% | 0.00% | 100.00% | 0.00% | 107.39 | >0.00001 | 124.42 | >0.00001 |
| BxC^-5^ |  |  |  |  |  |  |  |  |  |
| BANM simulation | 3.40% | 22.82% | 0.00% | 73.79% | 26.21% |  |  |  |  |
| BDS simulation | μ=2.9%  [0.7, 6.2] | μ=14.7%  [10.6, 24.8] | μ=0%  [0,0] | μ=82.5%  [69.9, 87.1] | μ=21.5%  [12.9, 30.1] | 13.11 | 0.002036 |  |  |
| day 1 | 0.00% | 0.74% | 0.00% | 99.26% | 0.74% | 191.79 | >0.00001 | 149.06 | >0.00001 |
| day 5 | 0.00% | 0.00% | 0.00% | 100.00% | 0.00% | 198.84 | >0.00001 | 144.39 | >0.00001 |
| day 15 | 0.10% | 0.77% | 0.00% | 99.13% | 0.87% | 229.71 | >0.00001 | 187.93 | >0.00001 |
| day 30 | 0.08% | 0.33% | 0.00% | 99.59% | 0.41% | 296.95 | >0.00001 | 234.26 | >0.00001 |

**Table S5.** Network vulnerability scores for donor communities, BANM and BDS simulations, and empirical coalescence outcomes across biotic dilution levels and time. For the BDS simulations, values are presented as the mean (μ) of 20 simulated networks, and the values in brackets represent the observed range (minimum–maximum) across those simulations.

| Treatment |  |  |  |  |  |  |
| --- | --- | --- | --- | --- | --- | --- |
| Donor A^-1^ | 0.045 | Donor B^-1^ | 0.037 | Donor C^-1^ | 0.034 |  |
| Donor A^-3^ | 0.020 | Donor B^-3^ | 0.060 | Donor C^-3^ | 0.020 |  |
| Donor A^-5^ | 0.010 | Donor B^-5^ | 0.033 | Donor C^-5^ | 0.032 |  |
|  |  |  |  |  |  |  |
|  | BADM simulation | BDS simulation | day 1 | day 5 | day 15 | day 30 |
| AxB^-1^ | 0.035 | μ=0.025 [0.008‚0.069] | 0.015 | 0.010 | 0.007 | 0.005 |
| AxB^-3^ | 0.020 | μ=0.062 [0.017‚0.107] | 0.022 | 0.043 | 0.018 | 0.019 |
| AxB^-5^ | 0.028 | μ=0.038 [0.019‚0.100] | 0.011 | 0.032 | 0.022 | 0.073 |
| AxC^-1^ | 0.079 | μ=0.020 [0.011‚0.045] | 0.015 | 0.048 | 0.014 | 0.031 |
| AxC^-3^ | 0.032 | μ=0.026 [0.017‚0.040] | 0.007 | 0.026 | 0.013 | 0.037 |
| AxC^-5^ | 0.021 | μ=0.025 [0.013‚0.050] | 0.042 | 0.026 | 0.036 | 0.049 |
| BxC^-1^ | 0.048 | μ=0.025 [0.011‚0.063] | 0.014 | 0.009 | 0.013 | 0.042 |
| BxC^-3^ | 0.013 | μ=0.028 [0.016‚0.084] | 0.042 | 0.024 | 0.064 | 0.037 |
| BxC^-5^ | 0.047 | μ=0.020 [0.009‚0.044] | 0.029 | 0.022 | 0.010 | 0.011 |

**Table S6.** Results of permutation-based ANOVA for distance-based redundancy analysis (db-RDA) using Euclidean distances and 999 permutations.

| **AxB** |  |  |  | **AxB** |  |  |  |
| --- | --- | --- | --- | --- | --- | --- | --- |
| RDA (stability ~ | Adj R^2^ | *F -* value _permutation_ | *P -* value _permutation_ | RDA (complexity ~ | Adj R^2^ | *F -* value _permutation_ | *P -* value _permutation_ |
| #OTU | 0.5170 | *F* = 12.78 | *P* = 0.005 | #OTU | 0.712 | *F* = 28.16 | *P* = 0.003 |
| Shannon | 0.5300 | *F* = 13.41 | *P* = 0.001 | Shannon | 0.694 | *F* = 25.95 | *P* = 0.005 |
| **AxC** |  |  |  | **AxC** |  |  |  |
| RDA (stability ~ | Adj R^2^ | *F -* value _permutation_ | *P -* value _permutation_ | RDA (complexity ~ | Adj R^2^ | *F -* value _permutation_ | *P -* value _permutation_ |
| #OTU | -0.071 | *F* = 0.27 | *P* = 0.818 | #OTU | 0.020 | *F* = 1.22 | *P* = 0.291 |
| Shannon | -0.074 | *F* = 0.24 | *P* = 0.824 | Shannon | -0.096 | *F* = 0.04 | *P* = 0.908 |
| **BxC** |  |  |  | **BxC** |  |  |  |
| RDA (stability ~ | Adj R^2^ | *F -* value _permutation_ | *P -* value _permutation_ | RDA (complexity ~ | Adj R^2^ | *F -* value _permutation_ | *P -* value _permutation_ |
| #OTU | -0.0372 | *F* = 0.61 | *P* = 0.554 | #OTU | -0.074 | *F* = 0.25 | *P* = 0.615 |
| Shannon | 0.0103 | *F* = 1.11 | *P* = 0.346 | Shannon | -0.042 | *F* = 0.56 | *P* = 0.452 |

**Supplementary Figures**

**Figure S1.** Boxplots showing the number of OTUs in donor and outcome communities across biotic dilution levels over time. Capital letters indicate statistically significant differences among donors and mixtures within each dilution level, whereas lowercase letters indicate significant differences among outcome communities across time and biotic dilution treatments (Tukey’s HSD, *P* < 0.05).

**Figure S2.** Boxplots showing the Shannon diversity index of donor and outcome communities across biotic dilution levels over time. Capital letters indicate statistically significant differences among donors and mixtures within each dilution level, whereas lowercase letters indicate significant differences among outcome communities across time and biotic dilution treatments (Tukey’s HSD, P < 0.05).

**Figure S3.** Network robustness (weighted and unweighted) across treatments and biotic dilution levels. Points represent mean robustness values, and error bars indicate 95% confidence intervals. Panels (A–C) show weighted robustness scores for AxB, AxC, and BxC, respectively, whereas panels (D–F) show the corresponding unweighted robustness scores for AxB, AxC, and BxC.

**Figure S4.** Network fragmentation across treatments and biotic dilution levels. Points represent mean fragmentation slopes, and error bars indicate 95% confidence intervals. Panels show fragmentation scores for AxB (A), AxC (B), and BxC (C).

**Figure S5.** Sensitivity of network properties to correlation threshold selection. (A) Spearman correlations between network trajectories across dilution levels and time points obtained using |*ρ*| ≥ 0.6 or |*ρ*| ≥ 0.8 and those obtained using the |*ρ*| ≥ 0.7 threshold used in the main analysis. Values within cells indicate Spearman’s *ρ*; significance is denoted as “***” *P* < 0.001, “**” *P* < 0.01, “*” *P* < 0.05, and “ns” *P* > 0.05. (B–D) Correlation threshold-response profiles for network degree, relative modularity, and vulnerability; lines connect the same network reconstructed at each of the three correlation thresholds.

**Supplementary References**

1. Dunne JA, Williams RJ, Martinez ND. Food-web structure and network theory: the role of connectance and size. Proc Natl Acad Sci U S A. 2002;99:12917–12922. <https://doi.org/10.1073/pnas.192407699>

2. Yuan MM, Guo X, Wu L, Zhang Y, Xiao N, Ning D, et al. Climate warming enhances microbial network complexity and stability. Nat Clim Chang. 2021;11:343–348. <https://doi.org/10.1038/s41558-021-00989-9>

3. Blondel VD, Guillaume JL, Lambiotte R, Lefebvre E. Fast unfolding of communities in large networks. J Stat Mech. 2008;2008:P10008. <https://doi.org/10.1088/1742-5468/2008/10/P10008>

4. Maslov S, Sneppen K. Specificity and stability in topology of protein networks. Science. 2002;296:910–913. <https://doi.org/10.1126/science.1065103>

5. Rillig MC, Antonovics J, Caruso T, Lehmann A, Powell JR, Veresoglou SD, et al. Interchange of entire communities: microbial community coalescence. Trends Ecol Evol. 2015;30:470–476. <https://doi.org/10.1016/j.tree.2015.06.004>

6. Dormann CF, Fründ J, Blüthgen N, Gruber B. Indices, graphs and null models: analyzing bipartite ecological networks. Open Ecol J. 2009;2:7–24. <https://doi.org/10.2174/1874213000902010007>

7. Herren CM, McMahon KD. Cohesion: a method for quantifying the connectivity of microbial communities. ISME J. 2017;11:2426–2438. <https://doi.org/10.1038/ismej.2017.91>

8. Kajihara KT, Hynson NA. Networks as tools for defining emergent properties of microbiomes and their stability. Microbiome. 2024;12:184. <https://doi.org/10.1186/s40168-024-01868-z>

9. Montesinos-Navarro A, Hiraldo F, Tella JL, Blanco G. Network structure embracing mutualism–antagonism continuums increases community robustness. Nat Ecol Evol. 2017;1:1661–1669. <https://doi.org/10.1038/s41559-017-0320-6>

10. Muggeo VMR. segmented: an R package to fit regression models with broken-line relationships. R News. 2008;8:20–25.

11. R Core Team. R: a language and environment for statistical computing. Vienna: R Foundation for Statistical Computing; 2020.

12. Deng Y, Jiang YH, Yang Y, He Z, Luo F, Zhou J. Molecular ecological network analyses. BMC Bioinformatics. 2012;13:113. <https://doi.org/10.1186/1471-2105-13-113>

13. Oksanen J, Simpson GL, Blanchet FG, Kindt R, Legendre P, Minchin PR, et al. vegan: community ecology package. R package version 2.6-2; 2022.

14. McMurdie PJ, Holmes S. phyloseq: an R package for reproducible interactive analysis and graphics of microbiome census data. PLoS One. 2013;8:e61217. <https://doi.org/10.1371/journal.pone.0061217>

15. de Mendiburu F, Simon R. agricolae: ten years of an open source statistical tool for experiments in breeding, agriculture and biology. PeerJ. 2015;3:e1404. https://doi.org/10.7717/peerj.1404

16. Xu S, Li Z, Tang W, Dai Z, Zhou L, Feng T, et al. MicrobiotaProcess: a comprehensive R package for managing and analyzing microbiome and other ecological data within the tidy framework. 2022. <https://doi.org/10.21203/rs.3.rs-1284357/v1>
